# Physic and Physicians: A Medical Sketch Book, Exhibiting the Public and Private Life of the Most Celebrated Medical Men, of Former Days; with Memoirs of Eminent Living London Physicians and Surgeons

**Published:** 1839

**Authors:** 


### Medico-Chirurgical Review,

### No. LXII.

[No. 22 of a Decennial Series.]

JULY 1, TO OCTOBER 1, 1839.

I. PHYSIC AND PHYSICIANS: A MEDICAL SKETCH BOOK, EXHIBITING THE PUBLIC AND PRIVATE LIFE OF THE MOST CELEBRATED MEDICAL MEN, OF FORMER DAYS; WITH MEMOIRS OF EMINENT LIVING LONDON PHYSICIANS AND SURGEONS. Two Volumes, 12mo. London, Longman, Orme, and Co. 1839.

II. BIOGRAPHICAL MEMOIRS OF THE MOST CELEBRATED PHYSICIANS, SURGEONS, &c. &c. By *Thomas Joseph Pettigrew*, F.R.S. &c. &c. Price 3s. each Part.

IT would appear that the Profession are taking a warm interest in the lives and fortunes of medical men. Their biography comes to us in many shapes, in Journals, in Transactions, in the monthly publication of Mr. Pettigrew, and in the volumes now upon our table.

At first sight such biography seems hopelessly barren. The practice of physicians and surgeons is but the application of a special art, and one man feels the pulse and looks at the tongue of a patient, much as any one else would do it. The great events which influence the fate and move the sympathies of nations do not turn on our actions—our science is unintelligible our professional feelings uninteresting to the mass—and our lives are only checquered with such events as are either common to all men, or wear that exclusive professional garb which shuts them out from public curiosity.

Nor is the public curiosity excited. Medical biography interests medical men almost alone, and judging from the sale of some recent works, it does not influence them very deeply. Yet it is a pleasant, as well as instructive contribution to the light literature of our profession, and something at least should be known by ourselves of the lives of those who have attained celebrity amongst us. It is astonishing how little *is* known. The names of our distinguished predecessors are familiar to us only through their works, and we are generally ignorant of the particulars of their career.

Every medical man who makes the least pretension to a cultivated mind, and who aims at being deemed a gentleman, should be fairly read in the history of physic, and tolerably conversant with the lives of its chief ornaments. To be neither is to pronounce the severest satire on his own art—to look on it as a thing of empirical experience, which one man practices much like another, and is insusceptible of the dignity of history. We consider this, however, a false as well as a derogatory notion. The history of medicine teaches these important facts—that the science of medicine like most other sciences has been of slow growth, and has shared the fortunes of philosophy—and that its progress, though checked, has never been really arrested by the errors and follies of its professors. These facts are well calculated to encourage us. They may render us easy on the score of quackery. That never has exercised, nor ever will, any seriously injurious influence on medicine. Truth has nothing to fear from fraud—a temporary cloud may veil her, but her sun is sure to shine again.

Medical biography touches our personal feelings. We all have had, or are having, our struggles for success—we can all feel for the triumph or the failure of genius or of industry—we can all admit the uncertainty of public favour, and many of us may esteem it an useful lesson to learn how *that* has been caught. To all these feelings biography appeals—the aspirations of ambition are awakened by it and gratified—and the life of an illustrious man is seldom perused without the reader being rendered wiser, if not better than before.

On these accounts we have devoted some former articles to Medical Biography, and, as the subject is far from exhausted, we shall yet devote some more.

"Physic and Physicians," is the produce, as its nameless author tells us, of a hint in Johnson's Life of Akenside. It rather gives light sketches of both than a connected history of either. The work is in fact a sketch-book, as the titles of its chapters will shew. They are thus intituled :—1, Antiquity of Physic, and Defence of Medical Men. 2, Eccentric Medical Men. 3, Early Struggles of Eminent Medical Men. 4, Celebrated Medical Poets. 5, Sketches and Illustrations of Medical Quackery. 6, How to get a Practice, or the Art of Rising in Physic. 7, Chonicles of Warwick Hall ; and the Medical and Surgical Luminaries of the olden time. 8, Mad-doctors and Mad-houses. 9, Literary and Scientific Medical Men. 10, Medical Emigration. 11, Army and Navy Surgeons, and East India Company's Medical Service. 12, Sketches of Eminent Living Physicians. 13, Sketches of Eminent Living Surgeons.

From these Chapters we may pick at random, and select or withhold what we please. Our object will be to instruct as well as to amuse. Can we begin with any thing more agreable than :—

### How to get a Practice, or the Art of Rising in Physic.

No doubt this is a *taking* subject. It has been recommended, says our author, to a young physician who wishes to get into practice, to start with a new theory. Attempting to prove that the blood does not circulate would ensure a great degree of notice, and prove highly beneficial to him. Were he to endeavour to prove the unwholesomeness of some favourite and common article of diet—the more startling and extraordinary the opinion the better—he would obtain an enviable degree of notoriety. He must be singular and eccentric in his manners ;—it is a matter of indifference whether he be brutal, or polished and courtier-like.

"There are two ways, my boy," said Radcliffe to Mead, when the latter was commencing practice, "for a physician to treat his patients—either to bully or cajole them. I have taken the former course, and have done well, as you see; you may, perhaps, take the latter, and, perhaps, do equally as well." Skill in pursuits not very consonant to medical ones, now and then has a great effect in procuring practice; it has occasionally been found to have been of great use to affect fox-hunting, boxing, &c. Singularity fills the general run of mankind with wonder, and from wonder to admiration the transition is obvious.

A physician, he continues, should never affect ignorance of the cause of a complaint; he should place it in the pancreas, or pineal gland, if he has no other local habitation ready at the moment. Of course he must answer every question. A lady once asked her apothecary from what substance castor-oil was made; he, (more *au-fait* with the slang of the ring than with the science of botany, a hat or beaver being by the fancy termed a *castor*,) unembarrassed, said that it was made from the *beaver!* The lady was satisfied, and, no doubt, considered her medical adviser a quick and sensible gentleman. A patient was one day very anxious to know how long she should be ill: "Madam," replied the physician, "that depends upon the duration of the disease." "I am much obliged to you, Doctor, for your information," was the patient's wise answer.—Never readily acquiesce in any thing your patient or the nurse should say. "My doctor," we recollect hearing an elderly lady observe, "always assents to whatever I say: I think he must be a great fool." A physician should never omit to take his fee, unless he makes a practice of refusing the fees of clergymen.

Here follow two stories.

"A physician of Montpellier was in the habit of employing a very ingenious artifice to bring himself into notice with the public. When he came to a town where he was not known, he pretended to have lost his favourite dog, and ordered the public crier to offer, with beat of drum, a reward of twenty-five louis to whoever should find it. The crier took care to mention all the titles and academic honours of the peripatetic physician, as well as his place of residence. He soon became the talk of the town: 'Do you know,' says one, 'that a famous physician has come here, a very clever fellow, of high academic honours, he must be rich, for he offers twenty-five louis for finding his dog!' *The dog was not found, but patients were.*

A poor physician, with plenty of knowledge and no practice, imparted his troubles to one of his friends. 'Listen to my advice,' says the other, and follow it. 'The Café de la Régence is in fashion; I play at chess there every day at two o'clock, when the crowd is thickest; come there too; do not recognize me; say nothing, but seem in a reverie; take your coffee, and always give the waiter the money wrapped up in rose-coloured paper, and leave the rest to me.'

The physician followed his advice—he was in daily attendance at the café, when it was most crowded—sipped his coffee in quietness, and paid as his friend directed him to do. His oddity was soon remarked. The question was frequently asked, 'who is that grave gentleman—he always appears absorbed in his own cogitations.' His kind friend said to the customers of the coffee-house, 'Gentlemen, do not think ill of this man, because he seems an oddity; he is a profound practitioner. I have known him these fifteen years, and I could tell you of some wonderful cures he has performed; but he thinks of nothing but his books, and never speaks except to his patients, which has prevented me from becoming intimate with him; but if ever I am obliged to keep my bed, he is the doctor for me.' The friend followed this course, varying the style of his panegyric from time to time, until all his auditors entertained a high opinion of the doctor's skill, and by this means he soon obtained an extensive practice."* 548.

It is a good thing to belong to some religious sect. Of course you are employed in it. The "*thee*" and "*thou*" of Dr. Fothergill, of London, was supposed to be worth £2000 a year to him at least.†

Dr. Mead was the son of a dissenting minister; and whenever he was called out of his father's church, which was often the case, the preacher would stop in the middle of his discourse, and say, "Dear brethren, let me offer up a prayer for the safe recovery of the poor patient to whom my son is gone to administer relief." Mead's father had as keen an eye to business as S. S. Hutchinson. The next is not a bad piece of advice.

"Be careful not to pronounce an opinion on any case, until you have gone through the usual routine of feeling the pulse, looking at the tongue, &c. An eminent court physician, visiting a noble lady from whose family he had received many shining marks of liberality and confidence, the following scene took place:—'Pray, doctor, do you think I might now venture on a slice of chicken, and a single glass of Madeira, as I feel very faint and low.' 'Most certainly, please your ladyship; I perceive nothing in the state of your pulse, or the appearance of your tongue, to forbid so reasonable an indulgence.' Her ladyship instantly rang the bell, and with more than usual peremptoriness of manner, desired the servant to order Sir ———'s carriage; then turning to the doctor, she addressed him nearly as follows:—'Sir, there is your fee, and, depend upon it, it is the last you shall receive from me, or from any of my connections with whom I possess any influence. I asked you a question, a serious question, Sir, to me, considering the very abstemious regimen to which I have so long submitted under your direction; and I think it full time to withdraw my confidence from a physician who delivers a professional opinion without any foundation; for, Sir, you must be perfectly aware that you neither felt my pulse, nor examined my tongue." 352.

In your instructions (we are still quoting) to your patients be particular in giving minute directions concerning diet. This has great effect on the minds of old women, especially if their maladies are, in a great measure, imaginary. Give a list of what is to be eaten at breakfast, dinner, and supper, and you may depend upon being often made the subject of conversation, and you will be considered a very "clever doctor." A physician, in large practice, brought himself into notice, by always recommending the *left leg* of a boiled fowl; and upon our attempting to persuade the invalid that the left leg possessed no peculiar virtue, she was quite indignant, and exclaimed, that "so sensible a physician must know better than you."

---

* "Coleridge tells a story of a lady who had a husband not overburdened with brains, but with sense enough to hold his tongue when in company. His taciturnity was noticed, and on the subject being mentioned to the wife, she mysteriously observed, 'Dear Sir, he is always thinking of Locke and Newton.'"

† "A Quaker apothecary meeting Dr. Fothergill, thus accosted him, 'Friend Fothergill, I intend dining with thee to-day.' 'I shall be glad to see thee,' answered the doctor; 'but pray, friend, hast thou not some joke?' 'No joke, indeed,' rejoined the apothecary, 'but a very serious matter. Thou hast attended friend Ephraim these three days, and ordered him no medicine. I cannot at this rate live in my own house, and must live in thine.' The doctor took the hint, and prescribed handsomely for the benefit of his friend Ephraim, and his friend Leech the apothecary!"

We hardly think it necessary to communicate any further the directions of our author. They are made up of the usual recipes, often given, occasionally followed, and *never* (we are inclined to think) successful. For instance, the young physician is told:—It is a great point gained if you can visit the opera frequently; and be sure to instruct the messengers, when the performance is over, to vociferate loudly for your carriage. This is an effectual way of making you known, as a London physician and a man of fashion. Be regular in your attendance at church; and instruct your servant to call you out occasionally during the service. When you first start in practice, it will be of service if you can persuade your *carriage friends* to call often at your house. Always contrive to have a coach standing at your door on Sunday, as it is sure to attract the notice of the people as they return home from church, and will lead the public to believe that you are a practising physician. We believe we may venture to say that no man ever did get into practice by such arts—and we are pretty sure that no man ever will. Indeed it is the dwelling on such things as these that makes us doubt if the author of these sketches belongs to the medical profession.

Whatever may be thought on this score, scepticism itself must own conviction on reading the following letter. It is addressed by Mead to a young physician, professes to give him advice on the best method of succeeding in practice, and is perhaps one of the severest satires upon physic that has seen the light in our days. The letter is long, but no one will regret reading it.

"That which gives me great hopes of you (Dr. Timothy Vanbustle, M.D.) is your resolution to go on, and to push into practice at all hazards. Monsieur De Rochf observes, that there is nothing impossible if we have but the resolution to take the right way to it. Besides, you know *audaces fortuna juvat;* and, therefore, above all things, let me as a friend advise you to take care of studying or endeavouring to know much in this way, since that will render you timorous and cautious, and consequently keep you back in your practice; besides that, the more you search the less you will be satisfied; and when arrived at the top of all, you may with Solon conclude, that all your wisdom (comparatively with real knowledge) is in knowing nothing. Whereas if you only *skim* the surface, you will go boldly on, and fancy your knowledge ten times more than what it really is. Thus then the great and principal thing you ought to be qualified with, is the *formula prescribendi;* for form is now the main chance, whether in law or physic, and without that, there is nothing to be done; this is the business, the *alpha* and *omega,* the all in all; some will succeed, and some won't; 'tis hit or miss, luck's all; you are paid, go which way you will. And now you are just arrived in town, without having had the benefit of establishing an acquaintance at Oxford or Cambridge, among the nobility, clergy, &c., and an absolute stranger here, without the assistance of dissenting teachers, relations, old women, nurses, children, or apothecaries; the first thing I advise you to do is to make all the noise and bustle you can, to make the whole town ring of you, if possible; so that every one in it may know that there is such a being, and here in town, too, such a physician. It signifies little which way it be, so it be done, and that your name be known and heard of, for that is half in half, since no one sends to consult him they have not heard of, that being a crime sufficient not to have been talked of; whereas, if accustomed to your name, you are a fit person to be called to the sick. Thus the famous R——f,*

* Radcliffe, we suppose.

'tis said, on his first arrival, had half the porters in town employed to call for him, at all the coffee-houses and public places, so that his name might be known. A very famous oculist has likewise freely told me, that he must starve, did he not frequently put himself in the public prints : but this is not so fashionable with physicians, ready to their company, or that which they think their company understands the best, or are otherwise so complaisant, as to talk to their friends of their interest, for I would suppose you have insinuated yourself into their friendship. Besides that, the very seeing you now and then, might put them in mind of that which they might otherwise forget. The old and the simple, the riotous, the whimsical, and the fearful, are your most proper company, and who will provide you with most business; there being far less to be got by the wise and sober, who are much more rarely ailing. But then you will, perhaps, tell me, that such like physicians will be the most proper to please and keep company with such, since *similis, simili gaudet*. If so, then I can only say, that those probably will stand the fairest for business; and if you are so wise or unwise, as not to ply, bend, truckle to their humours, I doubt you will be in danger of having less business : or, otherwise, if you would still continue, and be esteemed very wise, sober, and grave, you should then learn most obsequiously to fawn and sooth man, woman, and child, since few else will thrive well, unless blessed with wit, in which case, they may be allowed a little more liberty. To make yourself known, the making friends for some public lectureship is not amiss, which serves for a feather in your cap, by which you become known, and so taken notice of as a fine fellow ; and then you have an opportunity of haranguing your auditory, which, though it should be mobbish or trifling, you gain your point. As to what you read or say, it matters not much; if from the more musty and ancient authors the better; if from the more modern, the more fashionable it will be: and thus consequently you will either be esteemed a very learned, or at least a very ingenious man. If you can be introduced to an hospital, your business is done for life, be your success what it will. If your wife should happen to mind business in her way, it will certainly also encrease your's, for many good reasons, as encreasing your friends and acquaintances. It will not be amiss to set up an equipage, to purchase a mountain of books, and add any thing by which you will acquire the reputation of being a learned and ingenious gentleman. Let your religious and political opinions swim with the tide, especially when fashionable. Let not your fingers be sacrilegiously defiled, but be very gentle in taking fees of the clergy, &c. People are generally employed in proportion to the manner they live in, especially if once a little known; for the employing of many artificers and tradesmen, &c. you may not only become more known, but they also support and employ you. Thus, if you get much, you must spend much; and if you spend much, you will readily get much, particularly if spent in a proper way, and once a little known. *Don Quevedo* is of opinion, that the best way to run into business is to run into debt, because your creditors will employ you to get paid :—as to putting this experiment in practice, I shall rather choose to leave it to your own natural genius to direct you therein, than much to persuade you thereto, since there may be danger, should it not succeed.

To these hints I must observe to you, that dancing and dressing well, are not such slight accomplishments to introduce a young physician into practice, as you may imagine, because it makes him acceptable to the ladies and *beau monde*: his fashionable gesture, and gentle manner of feeling a pulse agreeably, is half the business; nay, that, and very little else, may, in time, for ought I know, go a great way towards an hospital, or other public employ. In fine, I shall now leave you ; may you live and brush on, so you may take the other ways to it.

I could mention you some, who got into business in physic, by writing poetry, some by divinity, others by politics, &c. But should you have an itching to make your name known by writing a book on physic, yet so customary, I would advise you to choose the subject by which you think you will get most money, or that which will bring you the most general business, as fevers, small-pox, &c. For in those, some must always live, some die, 'tis a hard matter to tell when right, or when wrong; write which way you will, 'tis disputable; but, certain it is, that the world in general readily conclude that you certainly understand that which you write about. The method of writing, if in your frontispiece you address not your book to some great man, is to club with some other physicians; and thus by way of letters, to commend each other's good practice, and to support and make each other famous. But above all things, take particular care, let the subject be what it will, that the words be well chosen, so as to make up an elegant and florid speech; since you have ten to one that mind the language more than the ideas, as Mr. Pope says:—

> 'Others for language all their care express,
> And value books, as women men, for dress;
> Their praise is still—'the style is excellent;'
> The sense they humbly take upon content.'

And next, then, I would advise you, whatever the subject be you write upon (if uncommon the better), rather to write, so as that no man can make any thing of it, so as neither to make downright sense, or nonsense thereof, than otherwise; because then none of the profession can well lay hold of you for any particular part; or if they should, there is room for you to defend it, being as easy to be understood one way as t'other. This is that method I commend, which Mr. Locke observes to be possible enough, for one to write a tolerable discourse of well-chosen and well-joined words, which nevertheless, on the whole, makes not up any real sense, or intelligible meaning. Thus I will suppose a man to write of sleep; now if I wrote in this manner, it is ten to one but that it will make all who read it fall asleep, and, consequently, what can be better said on the subject.

The last thing I advise you to do, is to get acquainted and cheerfully to keep company with all old women, midwives, nurses, and apothecaries, since these will still be entertaining you in the way of your business, and as the old ladies, &c. are most subject to ailings, so they will still be acquainting you with the same; and consequently, you are to make the most of it, and never to neglect or make slight of the least complaint; and thus you will gain the reputation of being both careful and skilful; whereas otherwise your care and skill may be suspected as well as your affectation." 360.

This letter bears in it the marks of seriousness, or we should have thought that Mead was bantering his protégé. It unquestionably affords a very sorry picture of the state of physic in those days, and proves its professors to have been little better than a set of hypocritical quacks. What a farce must men like Mead have considered medicine, when they looked on a deep acquaintance with it as an evil, because it would make a man timid. And what a commentary on authorship does Mead supply us with. Yet he was himself an author! Can we wonder at the false facts which oppress us, when we read such confessions from men of respected names? Every body knows that Sydenham, when asked what books a young physician should study, named "Don Quixote." He was of much the same opinion as Mead. He had as great a contempt, we are told, for physic, as he had for physicians, avowing it as his opinion, that the whole art might be written on a sheet of paper. Yet, it may be doubted, whether a more luminous lesson was ever given, than his declaration that, when a young practitioner, he possessed twenty remedies for every disease, and before the end of his career, he found twenty diseases, for which he had not one remedy.

A curious anecdote, by the bye, is given of Radcliffe's patronage of Mead.

" 'A little behind my house,' says Dr. Cumming, 'lies Carshalton, at which place, in days of yore, I have been informed that Dr. Radcliffe and the great Dons in his day, held an hebdomadal meeting, sacred, not to Æsculapius, but to Bacchus. To admit a young physician to one of these meetings, was deemed a distinguished honour; for no one was asked unless he seemed likely to prove conspicuous. When Dr. Mead was young, and just beginning to be talked of, he was asked to Carshalton; the object was to make him drunk, and to see the man: this design he suspected, and carefully avoided to fill a bumper when the sign was given.

'Mecum sæpe viri cum vino pellite curas:'

And he so managed as to see all the company retire under the table, except Radcliffe and himself; and the former was so far gone as to talk fast, and to shew himself affected by the potations. '*Mead,*' said he, 'will *you succeed me?*' '*It is impossible,*' replied the polite Mead; '*you are Alexander the Great, and no man can succeed Radcliffe: to succeed to one of his kingdoms is the utmost of my ambition.*' Radcliffe, with all his bluntness, was susceptible of flattery when delicately dressed up, and this reply won his heart. '*I will recommend you, Mead, to my patients,*' said he; and the next day he did Mead the honor to visit him in town, when he found him reading Hippocrates. Radcliffe with surprise asked, 'Do you read Hippocrates in the original Greek?' 'Yes,' answered Mead, respectfully. '*I never read it in my life,*' said the great Radcliffe. '*No!*' replied Mead, '*You have no occasion, you are Hippocrates himself!*' This did the business for Mead, and it completely gained the blunt Radcliffe; and when he did not choose to attend patients, he recommended Mead, who from that moment rapidly rose in his profession. 'This,' says Dr. Lettsom, 'I heard ten years ago from old Dr. Mounsey of Chelsea, who was one of the party; and since Crespigny, of Camberwell, told me the anecdote of this drinking party.' " 96.

But to return to Mead's letter and the art of getting practice. Extravagant as it may seem, and a burlesque on success in the profession, it was not inapplicable to the state both of medicine and society in those days. The prototype of Bob Sawyer then wore a wig and upraised a gold-headed cane. The ingenious Bob was forestalled by many an old physician. For example:—

" Dr. Edward Hans (afterwards Sir Edward Hans) having acquired an Oxford reputation, left the University, and settled in London as Dr. Radcliffe's rival. Dr. Hans was an excellent scholar, and a good chemist and anatomist. On his arrival in town, he set up a very spruce equipage, and in this way he endeavoured to attract the eyes of the public and obtain practice; but he found he was unable to compete with Dr. Radcliffe. Dr. Hans now had recourse to a stratagem; and, to get into repute, ordered his footman to stop most of the gentlemen's chariots, and inquire whether they belong to Dr. Hans, as if he was called by a patient. Accordingly the fellow, in pursuance of his instructions, stopped every coach from Whitehall to the Royal Exchange; and even went into Exchange Alley, and entering Garraway's Coffee-house, made the same interrogataries above stairs and below. At last Dr. Radcliffe, who was usually there about 'change time, and being seated at a table with several apothecaries and surgeons, cried out when Hans' footman asked, in breathless anxiety, for his master—' Dr. Hans is not here, what do you want with him?' The footman replied, that such and such a lord was taken ill. 'No, no, friend,' said Radcliffe, 'you are mistaken, the doctor wants those lords.' " 83.

There are some amongst us, who, alarmed at the progress of knowledge, and averse to those exertions which are absolutely indispensable to keep us in the foremost rank, are ever, like the smoke of a steam-boat pointing backwards, and are full of the real science, the dignity, and honour of the old physicians. Mead's letter has brushed the dust from these cobwebb'd pictures of respectability, and convinced us that the keeping and the colouring are but shabby, when stripped of the mystery that time and obscurity have thrown around them.

The fact is that the more medicine becomes a science, the less it will be practised as an art. In the hands of Sydenham, of Radcliffe, and of Mead, it was, as the latter justly described it, a thing of "hit or miss." Not knowing the real value of symptoms from their ignorance of morbid anatomy, the same remedy was applied to very different disorders, and the consequence was, of course, uncertainty and confusion.

### II. Illustrations and Sketches of Medical Quackery.

We have a Chapter on this score. We do not mean to argue the question of Quackery just now, but a few specimens of it may amuse.

The quack watchmaker was not a bad prototype of the quack doctor. A man's watch having stopped, he took it to a mechanic in order to ascertain the nature of the defect, and to have it rectified. The watchmaker armed his eyes with a microscope, and, after exhausting his customer's patience, for a considerable time being, as he thought, very sapiently occupied in examining the machinery to discover the disorder, observed that he could do no good to the watch without taking it all to pieces. It was carried to another, who, a good deal to the surprise of the owner, discovered, and honestly told him, that he *had only forgotten to wind it up!*

Pliny hit off, in an age of quackery, the reason of the success of quackery. Minus credunt quæ ad suam salutem pertinent, si intelligunt. The mystery is the thing.

The Chili doctors have not a bad way of *raising the wind.* "They blow around the beds of their patients, to drive away diseases; and, as the people in that country believe that physic consists wholly in their wind, their doctors would take it very ill of any person who should attempt to make the method of cure more difficult." So says Zimmerman, and he does not *puff* the Chili faculty.

We all know how the Quack's attestations of cures tell. The late Lord Gardestone, himself a valetudinarian, took the pains to inquire for those persons who had actually attested marvellous cures, and found more than two-thirds of the number died very shortly *after they had been cured.*

Yet the palmy days of Quackery have passed away, for where is the mountebank doctor at the fair? Our rustics have grown knowing and Katerfelto has shut up shop. The fact is that the mountebank has got a private house, and his clowns are noble lords and courtly dames. Hodge does not depose that St. John Long extracts mercury from a man's head. The illustrious peer who affirmed that, was not so inconsistent as might possibly be imagined. Knowing how much lead was contained in his own head, he might rationally suppose the presence of other metals in the heads of other persons.

The following account of the last Quack Doctor who exhibited, may be interesting. The famous Dr. Bossy was the man, and he has ceased to cure the incurable for fifty years.

"Every Thursday his stage was erected opposite the north-west colonnade of Covent-garden. The platform was about six feet from the ground, was covered, open in front, and was ascended by a broad step-ladder. On one side was a table, with a medicine chest, and surgical apparatus, displayed on a table with drawers. In the centre of the stage was an arm-chair, in which the patient was seated; and before the doctor commenced his operations, he advanced, taking off his gold-laced cocked-hat, and, bowing right and left, began addressing the populace which crowded before his booth.

The following dialogue between Dr. Bossy and a patient, we give verbatim, as it will afford the reader a characteristic specimen of one of the customs of the last age: it should be premised that the doctor was a humourist.

An aged woman was helped up by the ladder, and seated in the chair; she had been deaf, nearly blind, and was also lame; indeed, she might have been said to have been visited with Mrs. Thrale's *three* warnings, and death would have walked in at her door, only Dr. Bossy blocked up the passage.

The doctor asked questions with an audible voice, and the patient responded —he usually repeating the response, in his Anglo-German dialect.

*Doctor.* Dis poora voman vot is—how old vosh you?

*Old woman.* I be almost eighty, sir; seventy-nine last lady-day, old style.

*Doctor.* Ah, tat is an incurable disease.

*Patient.* O dear, O dear! say not so—incurable! why, you have restored my sight—I can hear again—and I can walk without my crutches.

*Doctor.* (smiling) No, no, good vomans, old age is vot is incurable; but, by the blessing of Gote, I vill cure you of vot is ilshe. Dis poora voman vos lame, and deaf, and almost blind. How many hosipetals have you been in?

*Patient.* Three, sir; St. Thomas's, St. Bartholomew's, and St. George's.

*Doctor.* Vot, and you found no reliefs? vot, none—not at alls?

*Patient.* No, none at all, sir.

*Doctor.* And how many professioners have attended you?

*Patient.* Some twenty or thirty, sir.

*Doctor.* O mine Gote! Three sick hosipetals, and dirty [thirty] doctors! I should vonder vot you have not enough to kill you twenty times. Dis poora vomans has become mine patient. Doctor Bossy gain all patients bronounced ingurables; pote wid de plessing of Brovidence, I shall make short work of it, and set you upon your legs again. Goode peoples, dis poora vomans vos teaf as a toor nails (holding up his watch to her ear, and striking the repeater). Can you hear dat pell?

*Patient.* Yes, sir.

*Doctor.* O den be thankful to Gote. Can you valk round this chair? (offering his arm.)

*Patient.* Yes, sir.

*Doctor.* Sit down, again, good vomans. Can you see?

*Patient.* Pretty so so, doctor.

*Doctor.* Vot can you see, good womans?

*Patient.* I can see the baker there (pointing to a mutton-pie man, with his board on his head).

*Doctor.* And vot else can you, good vomans?

*Old woman.* The poll-parrot there (pointing to Richardson's Hotel). "Lying old Bitch," screamed Richardson's poll-parrot. All the crowd shouted with laughter. Dr. Bossy waited until the laugh had subsided, and looking across the way, significantly shook his head at the parrot, and gravely exclaimed, laying his hand on his bosom, ''Tis no lie, you silly pird, 'tis all true as is de gospel." 320.

Even the glory of the bone-setters is departing. In another century we fear they will be no more, unless the great Mr. Radley succeeds in establishing his anti-splint practices, when bone-setters will be consulted properly enough.

But at present these worthies must sigh for the days of "Mother Mapp." She drove from Epsom, her residence, once a week to the Grecian Coffee-house in a coach and six with out-riders! It was in one of these journeys, passing through Kent-street in the Borough, that being taken for a certain woman of quality from the electorate in Germany, a great mob followed, and bestowed on her many bitter reproaches, till madame, perceiving some mistake, looked out of the window, and accosted them in this gentle manner, "D— your bloods, don't you know me? I am Mrs. Mapp, the *bone setter*!"

Out of the many absurd anecdotes of quackery we find before us, we shall take but one more, because the College of Physicians plays a ridiculous part in it. The college, which has rarely given itself the trouble of interfering with quacks, determined to shew its power and avenge the cause of legitimate physic on the devoted head of Dr. Brodum. We may well conceive the resolves that must have swelled the bosoms of the censors, and the anxious expectation that pervaded the world of quacks and doctors. Brodum was summonsed. He was punctual to the appointed time, and being acquainted only with the Hebrew and English languages, the president condescended to address himself in the latter. The Doctor replied, that he did not attend patients at their own homes, that he prescribed only his own medicine, for which he had obtained His Majesty's royal patent, and that, in difficult cases, he always recommended Sir Lucas Pepys, or some other able member of the college. The president observed that he did not wish to interfere with his nostrum, but that they required him to drop the title of Doctor, and put *Mister* Brodum instead of Doctor on his door, and to desist from taking fees. The Doctor replied, that all his fees came in letters from patients residing far beyond the limits of their jurisdiction; but if they thought he was encroaching on their privileges by having his letters of consultation addressed to his house in London, he would request his patients to direct to him at a place beyond their jurisdiction. As to his styling himself Doctor, he ventured to say that he had a right to do so, having a diploma from the Mareschal College of Aberdeen. "I suppose," observed a censor, "a purchased diploma." "I don't know," replied the Doctor, "what you mean by a *purchased* diploma; I suppose all physicians, who possess a diploma, paid for it. I should not have had a diploma had I not been considered worthy of it; and, as to my nervous cordial, Dr. Warren, and other learned members of your college, have done me the honor to take it, and recommend it!!" To the question, "How long did you reside at Aberdeen?" the Doctor replied, "I have never been there." He obtained his diploma on a certificate signed by Dr. Saunders, who was then one of the ruling members of the College of Physicians! The quack doctor refused to remove his brass plate from his door, and the college declined to carry their threat to remove it, into execution. The Mareschal College, on being apprized of the imposition, came to the resolution of prosecuting Dr. Saunders for a fraud; and had not his friends exerted themselves, it would have been put in force.

This scene and its results must have been highly flattering to the college and to the profession, and not a little diverting to Brodum and the public. The quack had much the best of it in every way. It was certainly a good joke to see the college prosecuting Brodum, when one eminent Fellow had procured him his Doctorate, and numbers of them had patronised his nostrum! This gave the college a sickener, and it never meddled with quacks again. Its powers, such as they were, they directed thenceforth against regular physicians.

### ECCENTRIC MEDICAL MEN.

Here, as we have hitherto done, we shall merely pick out a few passages containing amusement or information.

*Dr. Mounsey*, physician to Chelsea Hospital, was the Abernethy of his day. When he had grown very old, his birth being a comfortable one, numerous applications were made to the Minister of the day for his place. The aspirants longed for the old Doctor's demise, and the court of Chelsea Hospital became a favourite lounge for those who thought that the house and situation would exactly suit them.

One day Mounsey saw, from his window, a physician accompanied by his friend, who were taking a survey of the spot. The friend was pointing out to the candidate the pleasant situation of the medical apartments, and enumerating the various advantages of the college residence. As Mounsey was fond of teasing, he immediately descended. A few words served for his introduction; when turning to the physician, he said,

"So, Sir, I find you are one of the candidates to succeed me!"

The physician bowed and proceeded:

"But you will be confoundedly disappointed."

"Disappointed!" said the physician, with quivering lips.

"Yes," returned Mounsey, "you expect to outlive me; but I discern from your countenance, and other concomitant circumstances, that you are deceiving yourself—you will certainly die first: though as I have nothing to expect from that event, I shall not rejoice at your death, as I am persuaded you would at mine."

This was actually the case: the candidate lived but a short time, and Dr. Mounsey was so diverted with checking the aspiring hopes of his brethren of the faculty, that whenever he saw a physician on the look-out he used to go down and confront him in the same manner. He did so to several, and what is extraordinary, his prognostications were in every instance verified. The medical speculators shrunk aghast from Chelsea; so that at the death of the doctor, the minister was not engaged by a single promise, nor had he for some time a single application for the place of physician to the college.

He remained singular to the last, bequeathing his body for dissection, an old velvet coat to one friend, and the buttons of it to another. In his will he inveighs bitterly against bishops, deans, and chapters; and leaves annuities to two clergymen who had resigned their preferment on account of the Athanasian doctrine. He wrote his own epitaph, after being much teased by visitors with *kind* inquiries for his health.

"Here lie my old bones: my vexation now ends;
I have liv'd much too long for myself and my friends.

As to churches and churchyards, which men may call holy;

'Tis a rank piece of priestcraft, and founded in folly,

What the next world may be, never troubled my pate;

And be't what it may, I beseech you, O fate!

When the bodies of millions rise up in a riot,

To let the old carcase of Mounsey be quiet."

*Dumoulin* was one of the most famous physicians of his time. His practice was great, but his avarice was greater. This almost swallowed up every other feeling—it certainly got the better of any personal apprehensions. He was once visiting with M. Sylva a man of high rank, who died in their hands. This sudden death, being quite unexpected, occasioned considerable consternation and murmur in the apartment, particularly in the anti-chamber, where the domestics allowed themselves to adopt the most licentious conversation, and even threatened the doctors with unpleasant consequences. M. Sylva, naturally timid, was alarmed, and communicated his fears to Dumoulin, saying, "By what door shall we escape?" Dumoulin having no fear but that of not being paid, replied, "*By the door where they pay,*" and intrepidly left the apartment, followed by Sylva, who trembled with fear. A great miser, having heard that Dumoulin far surpassed him in saving knowledge, waited on him one winter evening about eight o'clock. He found him sitting, illuminated, or rather darkened, by the smoky light of a single lamp. On entering, he said to him, "I have heard that you are one of the greatest economists existing; I also am so inclined, but conscious of my inferiority, I should be happy to become your pupil on this point." "Is that all?" replied Dumoulin, "Sir, be seated;" in saying so, he extinguished the lamp. "There is no occasion for light to show us how to talk, it only produces inattention. Well then what is your object?" "Sir," cried the stranger, "the lesson of economy I have already received is enough. I shall always remain a scholar in respect to you. I shall endeavour to profit by the lesson I have received," and so withdrew in the best way he could in the dark.

*Sir Richard Jebb*, a physician to St. George's Hospital, cured the Duke of Gloucester of a dangerous illness. The King made him a Baronet, and appointed him his Physician Extraordinary. His Majesty, being indisposed one day, desired him to be sent for; but being told that it was etiquette to employ the physician in ordinary, he said, "Don't tell me of your 'ordinaries' or 'extraordinaries;' I will have Jebb."

Sir Richard's manners were *rather* bluff. Being called to see a patient, who fancied himself very ill, Jebb told him candidly what he thought, and declined prescribing, thinking it unnecessary. "Now you are here," said the patient, "I shall be obliged to you, Sir Richard, if you will tell me how I must live, what I may eat, and what not." "My directions as to that point," replied Sir Richard, "will be few and simple. You must not eat the poker, shovel, or tongs, for they are hard of digestion; nor the bellows because they are windy; but any thing else you please."

Nothing seems to have made him swear more than the eternal question, "What may I eat?" "Pray, Sir Richard, may I eat a muffin?" "Yes, madam, the best thing you can take." "O dear! I am glad of that. But, Sir Richard, you told me the other day, that it was the *worst* thing that I could eat!"

"What would be proper for me to eat to day?" says another lady. "Boiled turnips." "Boiled turnips!" exclaimed the patient, "you forget, Sir Richard, I told you I could never eat boiled turnips." "Then, madam, you must have a d——d vitiated appetite."

But he got, now and then, a Rowland for his Oliver. He once observed to a patient to whom he had been very rude, "*Sir, it is my way.*" "Then," replied the patient, pointing to the door, "I beg you will make *that your way.*"

Yet he appears to have been a mean-spirited fellow at bottom. What could be more snivelling than the following behaviour?

"I remember," says Dr. Lettsom, speaking of Jebb, "thy speech, 'Good God! Dr. Lettsom, Lord ———, whom I cured a year ago, has employed, lately, another physician.' 'Well, Sir Richard, you have more than you can do.' 'I know it, but this case has grieved me, incessantly, for a fortnight.' But when His Majesty turned away poor Jebb, and substituted in his place Sir George Baker, he never recovered from the blow. He left Great George Street, and pretended to retire to Lambs Conduit Street. But when the measles brought him again into the Royal Family, he was so agitated, that instead of sleeping at Windsor, he got up twelve times each night, was hurried, confused, tortured about the event, till at length, without apparent danger, he sent for Baker, to consult. After the termination of the measles, his own debility ensued, which he injudiciously increased by venesection; the effects of which no cordial could remove. But, poor fellow! a little before he died, the queen, in a letter, written by a German lady, inquired after his state of disease. This letter rekindled the expiring flame; he grasped it, and never parted with it, till life parted with his poor emaciated and weather-beaten frame!" 72.

Such abject conduct is too common in those who have basked in the sunshine of courts. Nor is it limited to physicians in ordinary. It was exhibited in the most marked manner by Wolsey and Bacon. If philosophy is so poor a solacer of court disgrace, physic need not blush at failing.

*Radcliffe*, the eccentric, daring, sceptical, and munificent physician, who contemned learning and left £40,000. to build a library, laughed at medicine, and founded two travelling fellowships for its promotion—and of whom Garth said that his building a library was much as if an eunuch should found a seraglio—was, after all, a little given to humbug. He always delivered his predictions in a tone of confidence. Having prophesied, with great accuracy, the fate of the Duke of Beaufort, he was always referred to as an oracle to decide disputed points of practice.

In Dr. Radcliffe's day great stress was laid on critical days. A slight cathartic on the *sixth* day of acute fever was considered fatal. A person having taken a gentle aperient on the sixth day, who laboured under an attack of acute fever, died. The person who prescribed the dose produced the recipe, and, in his own justification, pleaded the mildness of the medicine. Radcliffe, whose judgment was referred to, after an examination into the nature of the case, acknowledged indeed the mildness of the dose, but in his own style pronounced, "*it was ill-timed; disturbed a crisis, and thereby killed the patient.*"

This was not amiss. The next trait tops it.

In the month of December, 1694, Queen Mary was seized with the small-pox, which the court physician found it impossible to cure. Dr. Radcliffe was accordingly sent for by order of the Council to give his opinion.

At the first sight of the recipes, without seeing her Majesty, he told them, "she was a dead woman; for it was impossible to do any good in her case, where remedies had been given that were contrary to the nature of the distemper; yet he would endeavour to do all that lay in him, to get her some ease." Dr. Radcliffe's efforts to relieve his royal patient proved unavailing, and the queen died.

Radcliffe's tongue was certainly not attuned to courtly airs, nor did he "gloze or fawn, or bow the knee," to royalty. At one period he was highly in favour with the Princess Ann, of Denmark; but he forfeited her good graces, by his unpardonable rudeness. The Princess having been taken ill, the doctor was sent for, but he did not make his appearance as soon as was anticipated, and another messenger was dispatched for him. When the symptoms of her highness's distemper were related to him, he declared that "her malady was nothing but the vapours, and that she was in as good a state of health as any woman breathing, could she but believe it." In consequence of this uncourtly language, he was dismissed from his attendance on the princess, and Dr. Gibbons was nominated as his successor.

And yet he could shew adroitness on the nonce. He was sent for to see the king, who had been seized with symptoms resembling the dropsy. Dr. R. found the king reading Sir R. L'Estrange's new version of "Æsop's Fables." After a little preliminary conversation, the Doctor requested His Majesty to allow him to look at the book he was reading. Upon opening the volume, the doctor read to the king the following fable, in these words:—

"Pray, sir, how do you find yourself?" says the doctor to his patient. "Why, truly," says the patient, "I have had a most violent sweat." "Oh! the best sign in the world," quoth the doctor. And then, a little while after, he is at it again; with a "pray how do you find your lady?" "Alas!" says the other, "I have, just now, such a terrible fit of horror and shaking upon me!" "Why, this is all as it should be," says the physician, "it shows a mighty strength of nature." And then he asks him, a third time, the same question. "Why," says the patient, "I am all swelled, as if I had the dropsy." "Best of all," quoth the doctor, and goes his way. Soon after this, comes one of the sick man's friends to him, with the same question, "How he felt himself?" "Why, truly, so well," says he, "that I am e'en ready to die, of I know not how many *good signs and tokens*." "May it please your Majesty," says Radcliffe, "your's and the sick man's case is the very same." He advised the king to abroad, and upon his return, Dr. R. was sent for again. In reply to some questions put by the doctor, the king, showing his swollen ankles, which formed a striking contrast with the rest of his emaciated body, exclaimed, "and what think you of these?" "Why, truly," said he, "I would not have your Majesty's two legs for your three kingdoms."

Radcliffe was a practical joker as well as a wit. The art of uroscopy, satirized by Butler, and ridiculous at that time when so little was really known of the urine, was much in vogue in Radcliffe's day.

"Provided with this infallible indication of disease, a foolish woman called upon the doctor, and dropping a curtsey, told him, that having heard of his great fame, she had made bold to bring him a fee, by which means she hoped his worship would be prevailed upon to tell her the distemper her husband lay sick with, and to prescribe proper remedies for his relief. 'Where is he?' cries the doctor. 'Sick in bed, four miles off.' Taking the vessel, and casting an eye upon its contents, he enquired of the woman what trade the patient was of; and learning that he was a bootmaker, 'Very well,' replied the doctor: and, having retired for a moment, to make the requisite substitution, 'take this home with you, and if your husband will undertake to fit me with a pair of boots by its inspection, I will make no question of prescribing for his distemper, by a similar examination.'" 85.

He had a decided turn for humour, and like many wits relished the talent in another. He had, for instance, a great objection to paying his bills. A pavior, after long and fruitless attempts to get his account settled, caught Dr. R. just getting out of his chariot, at his own door, in Bloomsbury Square, and demanded the liquidation of his debt. "Why, you rascal," said the doctor, "do you pretend to be paid for such a piece of work? Why, you have spoiled my pavement, and then covered it over with earth to hide your bad work." "Doctor," said the pavior, "mine is not the only bad work that the earth hides." "You dog, you," said Radcliffe, "are you a wit? You must be poor—come in, and you shall be paid."

He was not devoid of feeling. "A lady of rank consulted him in great distress about her daughter, and the doctor began the investigation of the case by asking, "Why, what ails her?" "Alas! doctor," replied the mother, "I cannot tell; but she has lost her humour, her looks, her stomach; her strength consumes every day, and we are apprehensive that she cannot live." "Why do you not marry her," said Radcliffe. "Alas! doctor, that we would fain do, and have offered her as good a match as ever she could expect." "Is there no other that you think she would be content to marry?" "Ah, doctor, that is that troubles us; for there is a young gentleman we doubt she loves, that her father and I can never consent to." "Why, look you, madam," replied Radcliffe, gravely, "then the case is this: your daughter would marry one man, and you would have her marry another. In all my books I find no remedy for such a disease as this."

He had an odd escape from matrimony. "Being pressed by his acquaintances to marry, he looked out for a wife, and at length fixed upon the daughter of a wealthy citizen of London, with whom it was agreed he should have £15,000 down, and a still larger sum on the demise of her father, whom, however, he soon found reason to address in the following terms: 'Miss Mary is a very deserving gentlewoman; but you must pardon me, if I think her by no means fit to be my wife, since she is another man's already, or ought to be. In a word, she is no better and no worse than actually quick with child, which makes it necessary that she be disposed of to him that has the best claim to her affections. No doubt you have power enough over her, to bring her to confession, which is by no means the part of the physician. I shall wish you much joy of a new son-in-law, when known, since I am by no means qualified to be so near of kin. Hanging and marrying I find go by destiny; and I might have been guilty of the first, had I not so narrowly escaped the last.'"

His death was accelerated by his love of the bottle, doubts of physic, and inopportune bluntness. The latter was peculiarly unlucky for him. "On the 28th of July, 1714, Queen Anne was seized with a severe fit of illness, which terminated her life. Radcliffe was sent for; but he sent word that 'he had taken physic, and could not come.' In a letter to a friend he states his reason for not obeying the summons. He says, 'I knew the nature of attending crowned heads in their last moments too well to be fond of waiting upon them without being sent for by the *proper authority*.—You have heard of pardons being signed for physicians before a sovereign's demise.—However, as ill as I was (his disease was the gout,) I would have went to the Queen in a horse-litter, had either her Majesty, or those in commission next her, commanded me to do so.'

Radcliffe, however, confessed that everything had been done to save her Majesty's life. In consequence of Radcliffe's apparent neglect of the Queen, the popular feeling ran very strong against him. He felt this very acutely. He did not survive the Queen many months. An early biographer says, that he fell a 'victim to the ingratitude of a thankless world, and the fury of the gout.' He died in November, 1714, in the sixty-fifth year of his age."

We think we need not say much of his Christian learning or piety, when we mention the following anecdote. A little before he died, he read the twentieth or thirtieth chapter of Genesis, and observed, "he found Moses a clever fellow; if he had known him a little sooner, he thought he would have read him through."

*Abernethiana.*—This seems inexhaustible. We shall take a morçeau or two which is new to us, though possibly stale to some of our readers.

*A sensible Woman.*—A female, who, consulting Mr. A., for an ulcer she had on her arm, was particularly asked, "what is the matter with you?" The patient immediately held up her arm, but did not utter a word. "Oh! oh! poultice it, and take five grains of blue-pill every night, that's all; come again in a week." The fee was presented, but refused; at the end of the week, the patient presented herself again, when the same pantomime took place, and the fee again refused. After a few more visits, Mr. A., on looking at the arm, pronounced it well, when the patient again offered a fee. "No," said Abernethy, "from *you* nothing will I receive, for you are the most sensible woman I ever saw, *You don't talk!!*"

*Abernethy's Courtship.*—While attending a lady for several weeks, he observed those admirable qualifications in her daughter, which he truly esteemed to be calculated to make the married state happy. Accordingly on Saturday, when taking leave of his patient, he addressed her to the following purport:—"You are now so well, that I need not see you after Monday next, when I shall come and pay you my farewell visit. But, in the mean time, I wish you and your daughter seriously to consider the proposal I am about to make. It is abrupt and unceremonious, I am aware; but the excessive occupation of my time, by my professional duties, affords me no leisure to accomplish what I desire, by the mere ordinary course of attention and solicitation. My annual receipts amount to £——, and I can settle £—— on my wife; my character is generally known to the public, so that you may readily ascertain what it is. I have seen in your daughter a tender and affectionate child, an assiduous and careful nurse, and a gentle and lady-like member of your family; such a person must be all that a husband could covet, and I offer my hand and fortune for her acceptance. On Monday, when I call, I shall expect your determination; for I really have not time for the routine of courtship." In this way, however, the lady was wooed and won, and the union proved a happy one in every respect."

*His Independence.*—A certain noble personage, who at that time enjoyed a situation of great responsibility in the sister kingdom, had been waiting for some time in the surgeon's ante-room, when seeing those who had arrived before him, successively called in, he became somewhat impatient, and sent his card in. No notice was taken of the hint; he sent another card—another—another—and another; still no answer. At length he gained admission in his turn! and full of nobility and choler, he asked, rather aristocratically, why he had been kept waiting so long?—"Whe—ew!" replied the professor; "because you did not come sooner, to be sure."

*His account of himself.*—"People come here," he often was heard to say, "to consult me, and they will torture me with their long, foolish, fiddle-dee-dee stories; so we quarrel, and then they blackguard me all about this busy town; but I can't help it."

*His kindheartedness.*—In the year 1818, Lieutenant D—— fell from his horse on a paved street in London, and fractured his skull and arm, whilst his horse trod on his thigh and grievously injured his limb. Mr. A. was the nearest surgeon, and he was sent for; he came, and attended daily. After the lapse of months, convalescence took place, leaving great debility, when Abernethy enjoined the adoption of shell-fish diet, at Margate. His grateful patient requested information as to the amount of his pecuniary debt, for professional aid and care. Abernethy smiled, and said, "Who is that young woman?" "She is my wife."—"What is your rank in the army?"—"I am a half-pay lieutenant."—"Oh, wait till you are a general; then come and see me, and we'll talk about it."

*His Lectures at the College.*—On his receiving the appointment of Professor of Anatomy and Surgery to the Royal College of Surgeons, a professional friend observed to him, that they should now have something new. "What do you mean?" asked Abernethy. "Why," said the other, "of course you will brush up the lectures which you have been so long delivering at St. Bartholomew's Hospital, and let us have them in an improved form?" "Do you take me for a fool or a knave?" rejoined Abernethy; "I have always given the students at the Hospital that to which they were entitled—the best produce of my mind. If I could have made my lectures to them better, I would certainly have made them so. I will give the College of Surgeons precisely the same lectures down to the smallest details; nay, I will tell the old fellows how to make a poultice." Soon after, when he was lecturing to the students at St. Bartholomew's, and adverting to the College of Surgeons, he chuckingly exclaimed, "I told the big wigs how to make a poultice!"

*His generosity.*—Some years before his death, as Mr. Abernethy was walking up Holborn, he overtook one of his pupils; as was his custom when he had once noticed intrinsic talent, he entered into familiar conversation with him, observing that he had missed him for some time in the dissecting-room. The young man, with tears in his eyes, told him he was involved in debt, and that his parents, overtaken like himself by the shafts of adversity, could not grant him the necessary supplies. "To what amount are you in debt?" "About £80, sir," answered the poor bankrupt. "Well," said Mr. A. "call at Bedford-row to-morrow morning at ten o'clock, and I will see what can be done for you." The young man was obedient to the wishes of his kind instructor, when a letter sealed up was put into his hand, on opening which he discovered a cheque for ninety pounds!

*His civility.*—A gentleman farmer, from a distant part of the country, either fancying there was some derangement in his system, or wishing, after having seen the other sights of the metropolis, to visit one of its principal lions, Mr. Abernethy, accordingly went to him. "Do you make a good breakfast?" inquired Mr. Abernethy. "Pretty good," answered the patient. "You lunch?" "Yes, I take luncheon." "Do you eat a hearty dinner?" "Pretty hearty." "You take tea, I suppose?" "Yes, I do." "And, to wind up all, you sup, I suppose?" "Yes, I always sup." "Why then, you beast," said Abernethy, "go home and eat less, and there will be nothing the matter with you."

### Eminent Medical Men.

Every man in the profession desires, of course, to be eminent—most men, when they enter it, have a kind of notion that they *may* be so—and all would like to know how to become so. On this important question, as on most others in physic, there is a little difference of opinion. Some think chance has a good deal to do with it—others lay all the merit on connexion—others (the minority) believe that desert is necessary. So far as facts go, there would seem to be some truth in each opinion, the one least sustained by evidence being, perhaps, the last. But, since teaching by example is the most instructive, and as, of all useful knowledge, the most useful is how to get on in the world, we shall endeavour to shew, from the pages of our author, how a few lucky fellows *have* got on, and what manner of men they were.

*Dr. Thomas Willis* flourished much in the year 1685. He was regular in his devotions, his studies, and visiting his patients; and his custom was to dedicate his Sunday fees to the relief of the poor. Willis and Lower first recommended the waters of Astrop, which were afterwards decried by the celebrated Dr. Radcliffe; the reason for which was, the circumstance of the people of the village insisting upon the Dr. keeping an illegitimate child, which was laid to him by an infamous woman of that place: upon this, the Doctor declared "that he would put a toad into their well," and accordingly cried down the waters, which soon lost their reputation.

*The advantages of looking sickly.*—Everybody knows that physicians are much chosen for their looks. People might à priori suppose that the Doctor who contrived to look freshest and youngest would be selected by patients; for, they might argue, "if this man keeps himself in good health, and wards off the assaults and larcenies of old age, he is the man for our money." But the fact is just the reverse. A lank, superannuated face is a fortune. Such a physiognomy *must* belong to a first-rate man. It is indicative of *so* much thought, and of *such* solicitude for his patients.

*Dr. Gideon Harvey* found what a good thing it was to look ill. About the latter end of King William's reign, there was a great debate who should succeed the deceased physician to the Tower. The contending parties were so equally matched in their interests and pretensions, that it was extremely difficult to determine which should have the preference. The matter was at length brought to a compromise; and Dr. Gideon Harvey was promoted to that office; for the same reason that Sixtus V. was advanced to the pontificate; because he was in appearance sickly and infirm; and his death was expected in a few months. He, however, survived not only his rivals, but all his contemporary physicians; and died after he had enjoyed his sinecure above fifty years.

*How Mead got into practice and kept it.*—The Doctor had sacrificed rather more freely to the jolly god than was consistent with a healthy exercise of his faculties, and as he was feeling his patient's pulse, his foot slipped, when he ejaculated—"Drunk!—yes, quite drunk!"—alluding to himself. The Duchess, imagining he had found out her complaint, which she was most anxious to conceal, told Mead, if he kept her secret, she would recommend him. He did so, and the Doctor rose to fame and opulence.

Mead had a duel, as everybody knows, with Woodward, and one version of it is funny. It is said that Mead and Woodward met, and both drew, and that Mead, not loving cold iron, retreated; when Woodward, making a false step, fell down. His antagonist then ran in, and standing over him, demanded if he would submit and ask his life. "If you threatened me with your *physic*, I might beg my life," said Woodward, "but I certainly shall not ask it for fear of your sword."

Mead dabbled considerably in the stocks. One day, prior to his visiting his patients, he received intelligence that the stocks had suddenly fallen. At this moment he was sent for, in a great hurry, to visit a lady who was represented to be very ill. Having considerable property in the funds, the news made so strong an impression upon his mind, that, whilst he was feeling the patient's pulse, he exclaimed, "Mercy upon me, how they fall! lower! lower! lower!" The lady, in alarm, flew to the bell, crying out, "I am dying! Dr. Mead says, my pulse gets lower and lower, so that it is impossible I should live!" "You are dreaming, Madam," replied the physician, rousing himself from his reverie; your pulse is very good, and nothing ails you: it was the stocks I was talking of."

During almost half a century Dr. Mead was at the head of his profession; which brought him in, one year, upwards of £7,000., and between £5,000. and £6,000. for several years. His rival was Dr. Friend, whose practice was principally confined to the members of the high church or Tory party, Mead was patronized by the Whigs. When Dr. Friend was confined, during the suspension of the Habeas Corpus Act, under a suspicion of being concerned in a plot for the restoration of the Stuarts, Mead was incessant in his endeavours to obtain his liberation. At length, being called to attend Sir Robert Walpole, he absolutely refused to prescribe for him unless Friend was released, and he succeeded in effecting his object.

A large party assembled at Mead's in the evening to congratulate Friend; and, upon his retiring with Arbuthnot, Mead took Friend into his closet, and there put into his hands a bag containing all the fees he had received from Friend's patients during his confinement, amounting to no less than 5,000 guineas.

A gentleman, who was troubled with a constant affection of his eyes, waited on Dr. Mead for advice. The doctor desired him to leave off drinking wine. In a few weeks the patient felt the good effects of the prescription, and waited on the doctor to thank him for his advice. He was not a little surprised to find him in a tavern, discussing a bottle of wine with a friend. "Well," said the patient, "I see you doctors do not follow your own prescriptions." To which Mead replied, "If you love your eyes better than wine, don't drink it; but as I love wine better than my eyes, I do drink it."

This is something like Abernethy's defence of himself. "I am often asked—'Well, but Mr. Abernethy, why don't you practice what you preach?' I answer, by reminding the inquirer of the parson and the sign-post; both point the way, but neither follow its course.'"

The following is not a bad, though certainly rather a shocking story.

"Walpole says, that Lady Sandon's influence over Queen Caroline arose from her being possessed of the secret of her Majesty's being afflicted with *hernia umbilicus*. This, from motives of delicacy, she had communicated to the mistress of the robes, Lady Sandon; she was even so imprudent as to conceal her disease from the medical men, who treated her for gout of the stomach. When the danger became imminent, concealment was impossible.

Dr. Sands, an accoucheur, suggested that a cure might be effected by the injection of warm water. Dr. Mead entered a most positive protest against the experiment. Sir Edward Hulse was the only court physician who approved of the operation. At the time when the operation was performed, every wish to keep her Majesty's malady a secret must have been abandoned: for the courtiers, both male and female, were assembled in the anti-chamber, waiting anxiously the event.

The intestine was burst in the operation, and Dr. Sands and Sir Edward Hulse saw that the Queen must inevitably die of a mortification within a few hours. The only question which then remained for the two physicians to consider was, how they might get out of the palace before the unfortunate issue was known. They determined to say that the operation had succeeded. As soon as the two physicians came out of the Queen's chamber, and announced their success, the old Duke of Newcastle, who was among those who waited in the anti-chamber, ran up to Dr. Sands and hugged him, exclaiming, 'You dear creature, the nation can never sufficiently reward you for having saved the life of the most valuable woman in the world!' The doctor struggled to get away, apprehensive that some of the ladies, who had gone in to the Queen after the physicians had left her, might come out, and disclose the truth."* 20.

How many doctors' reputations seem to get wrecked at court! Royal bowels are ticklish things to meddle with.

It has been well said of Mead, that "of all physicians who had ever flourished, he gained the most, spent the most, and enjoyed the highest favour during his life-time, not only in his own, but in foreign countries."

*Dr. Cadogan* was a great shot in 1660. He married a rich wife, but the

---

* Nichol's "Recollections of Geo. III." vol. i. p. 369, and Cox's "Life of Walpole."

lady had a suspicion on her mind, that the doctor would one day poison her with his physic in order to get her out of his way, and feeling ill, on one occasion, she exclaimed that she was poisoned. "Poisoned!" said the doctor to a number of his wife's friends who were present, "how can that possibly be? Whom do you accuse of the crime?" "You," replied the indignant wife. "Gentlemen," said the doctor, with considerable *nonchalance*, "it is perfectly false. You are quite welcome to open her at once, and you will then discover the calumny."

*Dr. Fothergill's* name is familiar even to the present generation. He was the son of quakers, and himself a quaker. Of irreproachable conduct in general, his subsequent life contrasted strongly with an incident of his youth. While he resided at Edinburgh he walked the length of High-street naked to the waist, and, in a fit of inspiration, denounced God's vengeance on the inhabitants. This must have been a paroxysm of fanatical delirium.

Many instances are mentioned of his character. A physician of eminence, who had long been on a friendly footing with him, being under difficulties, and having a wife and several children to support, mentioned his distress to him, when, to his great satisfaction, Dr. Fothergill presented him with a draft upon his banker for £1,000.

When he paid his last visit to patients in decayed circumstances, it was not unusual with him, under the appearance of feeling the pulse, to slip into their hand a sum of money, or a bank-note. In one instance this mode of donation is said to have conveyed £150.

Such generosity was, perhaps, not the less meritorious because the doctor could afford it. We often find that the disposition to give is by no means in the ratio of the receipts. The Doctor, however, could afford it. Since his death it appears from his memoranda, that for the last twenty-five years his fees averaged £6,700 per annum. The fortune which he left was computed at £80,000. During the prevalence of the influenza, in the year 1775-6, he is said to have attended sixty patients a-day, and his profits were then estimated at £8,000 a year.

At the commencement of the American war, Mr. Grenville, then in power, wishing to know how the quaker colonists stood affected, sent a messenger to Dr. Fothergill, intimating that he was indisposed, and desiring to see him in the evening. The doctor came, and his patient immediately drew from him the information he wanted on the popular topic of American affairs. The conversation lasted through a large portion of the evening, and it was concluded by Mr. Grenville saying, he found himself so much better for the doctor's visit, that he would not trouble him to prescribe. In parting, Mr. Grenville slipped five guineas into the doctor's hand, which Fothergill surveying, said with a dry, arch tone, "At *this* rate, friend, I will spare thee an hour whenever you please to send for me!"

As Fothergill exhibited no commanding talent, enjoyed no powerful connections, and had not, so far as we know, any singular strokes of good fortune, it may naturally be asked, to what he owed his success. We will not pretend to answer the question directly, but we do not doubt that his manners and conduct helped him materially. "He possessed," writes Dr. Cuming, "a greater purity of manners, more self-government, and more absolute command of his passions, than any man I ever knew, who was constantly engaged in business, and a continued intercourse with the world. After saying thus much, I may be allowed to remark, that there was in his manner a *perpendicularity*, a certain *formality*, and *solemnity*, which checked, in some measure, the approach of strangers. He generally wore, indeed, on his countenance a smile—it was a smile of benignity and philanthropy, and, to his patients, it was a *hope-inspiring smile.*

> 'Seldom he laugh'd, and laugh'd in such a sort,
> As if he mock'd himself, and scorn'd his spirit
> That could be mov'd to laugh at any thing.' "

At first sight it would appear that the most opposite manners have succeeded equally in medical men, and that the rude and the courtly, the austere and the jocose have been pretty evenly esteemed. Ability will make any kind of manner tell—for it will affix upon each its stamp. But cæteris paribus, we are inclined to think that a certain amount of stateliness, a habitual reserve, and a solemn gravity, are the best manners for a physician. If he has genius and knowledge they acquire weight—if he has not, their deficiency is hid by such deportment. No doubt the humbug of a "sad and learned" countenance, does not pass quite so satisfactorily for wisdom as in former days; but it has still no slight effect, and we know a doctor or two, in this town, who could tell us so.

Fothergill is mixed up in a painful passage of Lord Clive's life. His lordship had been living for some time at Bath, under a regimen for reducing the enormous quantity of opium which he had gradually brought his constitution to bear; and when this object was in a great degree effected, the physicians absolutely forbad his taking the waters, and advised his return to London. On his arrival there, Dr. Fothergill, whom he immediately consulted, blamed him very much for the course he had adopted, and advised his immediate return to Bath, strongly recommending the waters of that place as the only means of relief. Thus buffetted about by his different physicians, and concluding from their conduct towards him that his case would not admit of any remedy, he resolved to obviate the lingering approaches of death by the fatal application of his own hand.

*Sydenham* had one or two odd notions. It is well known that he recommended young physicians to read Don Quixote, as *the* book of physic. Another crotchet was this. He had such confidence in exercise on horseback that in one of his medical works he says, "that if any man was possessed of a remedy that would do equal service to the human constitution with riding gently on horseback twice a day, he would be in possession of the philosopher's stone."

Sydenham died of the gout; and in the latter part of his life is described as visited with that dreadful disorder, and sitting near an open window on the ground floor of his house, in St. James's Square, respiring the cool breeze on a Summer's evening, and reflecting with a serene countenance, and great complacency, on the alleviation to human misery that his skill in his art enabled him to give. Whilst this divine man was enjoying one of these delicious reveries, a thief took away from the table, near to which he was sitting, a silver tankard filled with his favourite beverage, small-beer, in which a sprig of rosemary had been immersed, and ran off with it. Sydenham was too lame to ring his bell, and too feeble in his voice to give the alarm.

*Baillie*, says our author, was rejected, like Armstrong and Mason Good, at the College of Physicians! We need hardly say that this is fudge, and the story applies to another man. But the story itself, and the Editor's addenda, may amuse :—

"He called the next day on Dr. Barrowby, who was one of the censors, and insisted upon his fighting him. Barrowby, who was a little puny man, declined it. 'I am only third censor,' said he, 'in point of age, you must first call out your own countryman, Sir Hans Sloane, our president, and when you have fought him and two senior censors, then I shall be ready to meet you.'

Many medical duels have been prevented by the difficulty of arranging the '*methodus pugnandi*.' In the instance of Dr. Brocklesby, the number of paces could not be agreed upon; and in the affair between Akenside and Ballow, one had determined never to fight in the morning, and the other that he would never fight in the afternoon.

John Wilkes, who did not stand upon ceremony in these little affairs, when asked by Lord Talbot, 'how many times they were to fire?' replied, 'just as often as your lordship pleases; I have brought a *bag of bullets and a flask of gunpowder with me*.'" 43.

*Doctors don't "die hard*."—When Dr. Woodville, the author of the "Medical Botany," was supposed to be dangerously ill, his friends called upon him and endeavoured to excite his hopes of recovery: "I am not so silly," said the doctor, "as to mind what they say; I know my own case too well, and that I am dying: a younger person with a better stamina, might think it hard to die, but why should I regret to leave such a diseased worn-out carcase as mine?" The carpenter with whom he lodged, had not been always on the best terms with Woodville. The physician said he should wish to let the man see that he died in peace with him; and as he had never much occasion to employ him, desired he might be sent for to measure him for his coffin. This was accordingly done, the carpenter came, and took the measure of the doctor, who begged him not to be more than two days about it, "for," said he, "I shall not live beyond that time;" and he did actually die just before the end of the next day.

Dr. Fordyce, when near his latter end, desired his youngest daughter, who was sitting by his bed-side, to take up a book and read to him; she read for about twenty minutes, when the doctor said, "Stop, go out of the room, I am going to die." She put down the book, and left the chamber to call an attendant, who immediately went into the bed-room, and found Fordyce had breathed his last.*

Dr. William Hunter, a short time before he died, rose from his bed to deliver an introductory lecture on the operations of surgery, in opposition to the earnest remonstrances of his friends. The lecture was accordingly delivered, but it was his last. Towards the conclusion, his strength was so much exhausted that he fainted away, and was finally replaced in his cham-

---

* We give these anecdotes as we find them. Many are probably, some palpably, untrue.

ber, which he had been so eager to quit. Turning to his friend Combe, in his latter moments, he observed, "If I had strength enough to hold a pen, I would write how easy and pleasant a thing it is to die."

This same Fordyce, who died so comfortably, lived strangely. He dined every day for more than twenty years at Dolly's chop-house. His researches in comparative anatomy had led him to conclude, that man, through custom, eats oftener than nature requires, one meal a day being sufficient for that noble animal the lion. At four o'clock, his accustomed hour of dining, the doctor regularly took the seat at the table always reserved for him, on which was placed a silver tankard full of strong ale, a bottle of port wine, and measure containing a quarter of a pint of brandy. The moment the waiter announced him, the cook put a pound and a half of rump-steak on the gridiron, and on the table some delicate trifle, as a *bonne bouche*, to serve until the steak was ready. This, was sometimes half a boiled chicken, sometimes a plate of fish: when he had eaten this, he took one glass of brandy, and then proceeded to devour his steak. When he had finished his meal, he took the remainder of his brandy, having during his dinner drank the tankard of ale, and afterwards a bottle of port! He thus daily spent an hour and a half of his time, and then returned to his house in Essex-street, to give his six o'clock lecture on chemistry. He made no other meal until his return, at four o'clock next day, to Dolly's. How those who declaim on our degeneracy and the fine old customs and fine old constitutions of our forefathers will reconcile hypothesis with fact, we do not know. For our own parts, we confess that we are so effeminate as to think the state of society depicted by those etchings of our predecessors, very properly put an end to.

*The Danger of resuscitating a Jail-bird.*—Dr. Glover went for a short time on the stage. He conceived the idea that many persons in a state of suspended animation might be restored to life. The doctor was so confident of his opinion being well-founded, that he laid a wager with a brother comedian, that the first malefactor who was executed he would restore to life. The bet was accepted, and a few days after the doctor had an opportunity of proving that he was right, on the apparently dead body of a man who was hanged for a robbery. He was, however, rather unfortunate in the choice of his subject; for the following day, the fellow having discovered the doctor's lodgings, and being introduced into the apartment where he was sitting, the resuscitated criminal, accosting the preserver of his life by the familiar appellation of "*Father*," said that as he had restored him to existence, it was his duty to support him as his son, and this he should expect him to do. The singularity of the application so amazed the doctor, that it was some time before he recovered his powers sufficiently to enable him to expel him *vi et armis* from the room. Nothing daunted by his reception, he visited the theatre that evening, and harangued the audience from the gallery, whilst the doctor was acting. Wherever the poor doctor went, his resuscitated friend followed him, demanding a settlement for life. At last Dr. Glover was compelled, in order to get rid of his *hopeful heir*, to offer to advance him a sum of money if he would leave the kingdom. This was accordingly agreed to.

*Dr. George Cheyne*, the celebrated author of "The English Malady," on coming up to London found the bottle companions, the younger gentry, and free livers to be most easy of access, or most quickly susceptible of friendship and acquaintance; nothing being necessary for that purpose but to be able to eat lustily, and to swallow down much liquor; but his health became much damaged, and he turned round to a milk and vegetable diet, under which he recovered. He died at Bath, in 1743, where he had been for a long period, in rather extensive practice. Among his patients, was the celebrated Beau Nash; who, on being asked one day, by Cheyne, if he had followed his last prescription, replied in the negative, adding, "If I had, doctor, I should certainly have broken my neck, for I threw it out of a two pair of stairs' window."

A patient, accompanied by Beau Nash, visited Dr. Cheyne, for the purpose of ascertaining the cause of a slight abdominal swelling, under which he was labouring. On examining the patient, the doctor pronounced the swelling to be occasioned by a collection of water, and that it would be necessary to be tapped. "It cannot be water," said the patient, "it may be wine." "No, no, my good fellow," said Nash, "if it had been wine, you would long before this have tapped it yourself."

Dr. Cheyne and Tadlow were exceedingly corpulent men, but the last was much the larger. Cheyne coming into a coffee-house one morning, and observing Tadlow alone and pensive, asked him what occasioned his melancholy. "Cheyne," said he, "I have a very serious thought come athwart me: I am considering how the people will be able to get you and me to the grave when we die." "Why," said Cheyne, "six or eight strong fellows may take me there at once, but it is certain that *you* must be carried there at *twice*."

The editor of these sketches subjoins the entire case of Colonel Townshend, which was detailed by Cheyne in his "English Malady." Most of us have heard of the case, but few have read the original and highly curious account of it, which we are accordingly tempted to subjoin.

"Colonel Townshend, a gentleman of excellent natural parts, and of great honor and integrity, had for many years been afflicted with a nephritic complaint which made his life miserable. During one of his attacks of illness he sent for Dr. Baynard and myself: we waited upon him with Mr. Skrine his apothecary. We found his senses clear and mind calm: his nurse and several servants were about him. He had made his will and settled his affairs. He told us he had sent for us to give some account of an odd sensation he had for some time observed and felt within himself; which was, that composing himself he could die or expire whenever he pleased, *by an effort of the will*, and, by another effort, could come to life again, which it seems he had sometimes tried before he had sent for us: we heard this with surprise, but as it was to be accounted for on no common principles, we could hardly believe the facts he related to us, much less give any account of it, unless he should please to make the experiment before us, which we were unwilling he should do, lest in his weak condition, he might carry it too far. He continued to talk very distinctly and sensibly for above a quarter of an hour, about this surprising sensation, and insisted so much in our seeing the trial made, that we were at last forced to comply. We all three felt his pulse first, it was distinct, although small and steady; and his heart had its usual beating. He composed himself on his back, and lay in a still posture for some time: while I held his right hand, Dr. Baynard laid his hand on his heart, and Mr. Skrine held a clean looking-glass to his mouth. I found his pulse sink gradually, until at last I could not feel any, by the most exact and nice touch; Dr. Baynard could not feel the least motion in his heart, nor Mr. Skrine perceive the least soil of breath on the bright mirror he held to his mouth: then each of us by turns examined his arm, heart, and breath, but could not by the nicest scrutiny discover the least symptom of life in him. We reasoned a long time about his odd appearance as well as we could, and all of us judging it inexplicable and unaccountable, and finding he still continued in that condition, we began to conclude that he had indeed carried the experiment too far, and at last were satisfied he was actually dead, and were just ready to leave him. This continued for half an hour. As we were leaving, we observed some emotion about the body; and, upon examination, we found his pulse and the motion of the heart gradually returning: he began to breathe gently and to speak softly, and after some conversation with him we left him. He afterwards sent for his attorney, added a codicil to his will, settled legacies on his servants, received the sacrament, and calmly and composedly expired about five or six o'clock that evening." 63.

The only rational mode of accounting for these phenomena seems to us to be this—the patient being much reduced and near death, had fits of what may be almost syncope. Finding this, he either imagined or attained a power of inducing one. Be this as it may, the case is a very strange, as well as a very famous one, and will repay perusal.

*Harvey's Treatment of the Gout.*—This great man was a martyr to the complaint. He would sit with his legs bare, even if it were frosty, on the leads of Cochaine House, where he lived for some time with his brother Eliab, or put them into a pail of water until he was almost dead with cold; and then he would betake himself to his stove. He was troubled with insomnolency, to cure which he would rise in the night, and walk about his chamber in his shirt until he began to shiver, and then he would return to his bed.

*Dr. John Friend* wrote the history of medicine during his confinement in the Tower, in 1675. Friend, like most others of his day, was generally mellow after dinner. He was once sent for in this state, to a family of consequence; but the family not choosing to trust to his prescription, sent for Dr. Mead, who came, and, after looking at what Friend had written, took the opportunity of paying him a very high compliment. "'Pon my word," said Mead, "if Dr. Friend wrote this when he was drunk, he prescribes better than I can do when sober."

The following is a curious anecdote of Bishop Newton—curious in more than one respect. The Bishop *bled* freely to his doctors, though this antiphlogistic treatment did not cure him. "He was seized with a violent pleuritic fever—his illness cost him seven hundred and fifty guineas for physicians, and his cure was at last effected by *small beer*. Dr. Hope, Dr. Swynsen, and other physicians from Stafford, Lichfield, and Derby, were called in, and had two hundred and fifty guineas of the money. Dr. Friend came down post from London, with Mrs. Pulteney, and received three hundred guineas for his journey. Dr. Broxholme came from Oxford, and received two hundred guineas. When these physicians, who were his particular friends, arrived, they found the case quite desperate, and gave him entirely over. They said every thing had been done that could be done.

They prescribed some few medicines, but without the least effect. He was still alive, and was heard to mutter, in a low voice, "Small beer," "Small beer!" They said, give him small beer, or anything. Accordingly a great silver cup was brought, which contained two quarts of small beer. They ordered an orange to be squeezed into it, and gave it to him. He drank the whole at a draught, and called for another. Another was given him, and, soon after drinking that, he fell into a most profound sleep, and a most profuse sweat for nearly twenty-two hours. In him the saying was verified, "*If he sleep he shall do well.*" From that time he recovered marvellously, insomuch that, in a few days, the physicians took their leave, saying that, "Now he had no want of anything but a horse for his doctor, and an ass for his apothecary."

*Dr. John Arbuthnot*, the friend of Swift and Pope, was Queen Ann's favourite physician. He was possessed both of wit and genius. When a young man, he attempted to settle as a physician, at Dorchester, a town remarkable for its healthy situation, a circumstance unpropitious to the profitable practice of physic. On quitting it, a friend met him riding post to London. "Where are you going, Arbuthnot?" asked his friend. "To leave your confounded place, for a man can neither live nor die there," replied Arbuthnot.

The Queen having asked him what the time was, he replied, bowing with much grace, "Whatever it may please your Majesty."

Arbuthnot has given some amusing instances of the impression made on the public mind by Gulliver's travels when they first came out.

"Dr. Arbuthnot says, that Lord Scarborough (who was no inventor of stories) told him, that he happened to be in company with a master of a ship, who said, that 'he was very well acquainted with Mr. Gulliver: but that the printer had make a mistake, for it was at *Wapping*, and *not at Rotherhithe*, that the Captain lived.'

In another place, Dr. Arbuthnot says: 'I lent Gulliver's Travels to an old gentleman to read; who, putting on his spectacles, went very deliberately to his map to look for '*Lilliput*.'

Mr. Fortescue, (afterwards Master of the Rolls,) when a lawyer on the Western Circuit, wrote a letter to Mr. Pope, in the year 1727, stating that 'One Lemuel Gulliver had a cause there, and lost it on the ill-reputation he had acquired of being a most notorious liar:' and an Irish judge told some person of Swift's acquaintance, very gravely, that he looked upon the whole of Gulliver's Travels, (*whatever other persons might think of them,*) to be one continued heap of improbable lies." 71.

*Dr. James Gregory*, the able physician, illustrious teacher, and accomplished wit, has been heard to declare that nearly one-third of the patients who consulted him never paid their fees. He was a man too independent in spirit to demand what he had a right to claim, and consequently he was much imposed upon.

"That the profession are much exposed to the tricks of dishonest men, the following anecdotes will testify.

Of the noted Cooke who died some years ago at Pentonville, many anecdotes have been related, of plans which he practised in order to cheat medical men into gratuitous advice. One of his modes was to attire himself in rags, and ape the pauper. Another was to procure a letter from some dispensary, and to attend there, which he did once for several weeks before he was found out to be a man of immense fortune.

On some occasions, however, he found himself obliged to seek advice in *propriâ personâ*, but even then he went upon a saving plan : at one time, having a complaint in his leg, he applied to a surgeon in his neighbourhood, a Mr. Pigeon, asking him how long it would take to make a cure. The surgeon answered, 'a month.' This alarmed the miser, who anxiously enquired what would be the medical demand; and on being told by the surgeon, who saw the complaint was trifling, that it would be a guinea, Cooke replied, 'a guinea! very well; but mark this—a guinea is an immense sum of money; and when I agree upon sums of such magnitude, I go upon the system of no cure no pay : so if I am not cured by the expiration of the month, I am to pay nothing!'

Mr. Pigeon accepted the terms; and by his diligence and surgical skill, would have closed the wound completely some days within the limited space; which the miser observing, and trembling for his gold, was determined to prevent, if possible; and actually procured an irritating plaister, which he put on, so as to extend the period of cicatrizing, which was then going on wholesomely; and was thus enabled, on the last day of the specified term, to show to the surgeon that the wound was still unhealed. The unhappy wretch, proud of his own infamy, had the hardihood to boast of it afterwards, under the cant phrase of 'plucking a pigeon.'

Even on his death-bed the ruling passion was still strong upon him; for having sent for several medical men, one only would attend; and he having for a few days sent in some medicine, the dying miser at length entreated him to say how long he thought he might live. The candid apothecary honestly said, 'perhaps five or six days;' when Cooke, collecting all his strength for the moment, and starting up in his bed, exclaimed, 'There! are you not a dishonest man, a rogue, and a robber, to serve me so?' The apothecary, in some surprise, naturally enquired how he deserved those epithets. 'How, Sir!' faintly uttered the dying miser, 'why, you are no better than a pickpocket, to rob me of my gold, by sending in two draughts a day, to a man that all your physic will not keep alive for six days! begone from my house, and never enter it again.'" 85.

Most men in the profession are fully sensible, theoretically and practically too, of the disposition on the part of people to impose upon them. The general practitioner finds his bills unpaid—the pure is bilked of his fee. We have, more than once, been presented with a farthing, neatly wrapped up, lest our feelings should be shocked. Coronation tokens, Victoria tokens, bad sovereigns, and bad shillings, are all serviceable in the practical joke upon "the doctor." We remember hearing a story of the late Dr. Pearson, which evinces his dislike to being trifled with on the subject of fees. A patient consulted him and received a prescription. He gave the doctor half-a-guinea. "A guinea is my fee," quoth Pearson. "Oh." The patient seemed inclined to let matters stand as they were. "Oh," said Pearson, "I have made an omission; allow me to look at the prescription again." The patient gave it him, and he, taking pen, ink, and paper, began to write another. "It is a half-guinea prescription you want, not a guinea one, I see. I'll give you one of that sort directly." We need not say that the additional half-guinea was forthcoming in a twinkling.

*Dr. Lettsom*, born a quaker within the Tropics, apprenticed to Abraham

Sutcliff, a surgeon-apothecary in Yorkshire, attended St. Thomas's Hospital for twelve months, and then returned to the West Indies, to take possession of some little property, which had been left him by his father. It consisted of a small portion of land, and fifty slaves: and although, at this period, Lettsom was only possessed of fifty pounds in the world, he, with a feeling of humanity which does him immortal honour, soon after his arrival at Tortola, emancipated the whole of the slaves; thus reducing himself, by an act of justice, to great poverty, at the age of twenty-three.

Yet, some way or other, Lettsom was a lucky dog, for, in five months after commencing practice at Tortola, his native place, he succeeded in amassing nearly £2,000; half of which he gave to his mother, and with the other half he returned to England, with the view of following in the footsteps of Fothergill. His next hit was to marry a lady of great wealth. Such a beginning might justify any subsequent ill-luck. But the quaker did not know what ill-luck meant. For a considerable time he maintained the first practice as a physician in the city of London. His professional emoluments were great. In 1783, he received £3,600; in 1784, £3,900; in 1785, £4,015; and, in 1786, £4,500.

Mr. Pettigrew observes, had he at this time taken all the fees that were presented to him, his receipts would have been double. In 1800 he received, in fees, £12,000. This was considerably more than Dr. J. Fothergill ever derived from his practice; his highest sum was £5,000 in one year.

Lettsom was a very charitable and humane man, and maintained the usual character of the profession for liberality and generosity.

"In 1782, he was sent for to visit an old gentleman, 74 years of age, who resided in the county of Essex. This gentleman had been a great American merchant; he had kept a princely house, and his heart was literally made up of generosity. The American war ruined him; but his creditors, valuing his upright character, permitted him to reside at his house in the country, with a genteel allowance, until his affairs could be settled. The protracted American war destroyed the prospect of retrieving his affairs; his allowance was, therefore, taken away. He fell sick, and consulted Dr. Lettsom. When the Doctor visited him, the gentleman said to him, pointing to his garden, 'Those trees I planted, and have lived to see some of them too old to bear fruit. They are part of my family; and my children, still dearer to me, must quit this residence, which was the delight of my youth, and the hope of my old age." 93.

The Doctor, upon quitting the apartment, left, enclosed in a letter, a check to relieve his immediate necessities. He also purchased the house, which was freehold, for £500, and gave it him for life.

An adventure occurred to Lettsom, very similar to one related of Rowland Hill. It is thus told in his own words.

"It was my lot, a few years ago, to be attacked on the highway by a genteel-looking person, well mounted, who demanded my money, at the same time placing a pistol to my breast. I requested him to remove the pistol, which he immediately did. I saw his agitation, from whence I could perceive he had not been habituated to this hazardous practice: and I added that I had both gold and silver about me, which I freely gave him, but that I was very sorry to see a young gentleman risk his life in so unbecoming a manner, which would probably soon terminate at the gallows: that at the best, the casual pittance gained on the highway would afford but a precarious subsistence; but that if I

could serve him by a private assistance, more becoming his appearance, he might farther command my purse; and, at the same time, I desired him to accept a card containing my address, and to call upon me, as he might trust to my word for his liberty and life. He accepted my address, but I observed his voice faltered; it was late at night; there was, however, sufficient star-light to enable me to perceive, as I leaned towards him on the window of the carriage, that his bosom was overwhelmed with conflicting passions: at length, bending forward on his horse, and recovering the power of speech, he affectingly said, 'I thank you for your offer; American affairs have ruined me;—I will, dear Sir, wait upon you.'

The man kept his word, and Lettsom finding, on enquiry, the account he gave of himself to be correct, after making an unsuccessful application, in his behalf, to the Commissioners for relieving the American sufferers, presented a memorial on the subject to the Queen, who, it is said, procured the man a commission in the army, and his name subsequently appeared on two occasions, in the Gazette for promotion, on account of good conduct." 96.

Lettsom determined to assail quackery, and went about the thing very valiantly. He prevailed on Phillips, the proprietor of the "Medical Journal", to allow him to insert, in every number of that work, an anonymous article on each of the advertising quacks of the country. Lettsom began with Brodum, the proprietor of the Nervous Cordial, and other furiously advertised preparations; and, without ceremony, charged him with killing thousands by their indiscriminate use; and, to undermine Brodum, stated that he had been a shoeblack at Copenhagen; a jew vender of oranges, and, finally, a footman to a mountebank.

All this might be partly true, but it could not be legally proved in a court of law. Brodum, to whom the costs of a suit were of no importance, set his attorney to work, and Phillips, the printer, and three or four venders, were served with actions for £5,000 damages.

Phillips called on Lettsom, and the whole college, one by one, to enable him to justify, but in vain; for not one could prove that Brodum had even a single swallower of his nostrums. The lawyers held consultations, and the ingenious Garrow was anxious to get his brother-in-law Lettsom, out of the scrape. In the mean time, Brodum's attorney pressed for proceedings. At length, the editor of a newspaper stept between Phillips and Brodum; the latter agreed to withdraw his actions, and submit to the costs, provided the author was given up; but, if not, then he expected all expenses to be paid without demur; that the author should whitewash him in the next Journal, under the same signature; and, further, that Phillips, and the newspaper editor, should dine with him. Lettsom gladly paid the two attorney's bills, amounting to £390. Phillips had a splendid dinner, and the next Medical Journal contained a high eulogium on the talents and virtues of Dr. Brodum! Such was the issue of Dr. Lettsom's campaign against quackery.

He was remarkable for his attention to cleanliness. The mention of this gives occasion to the editor to introduce an anecdote which makes a fair addition to the stock of medical *bon mots.*

"This reminds us of an anecdote of an American physician, whom a lady consulted about her child's precious health. Among other things, she enquired if he did not think the *springs* would be useful. 'Certainly, Madam,' replied the Doctor, as he looked at the child, 'I have not the least hesitation in recommending the springs—and the sooner you apply the remedy the better.' 'You really think it would be good for the dear little thing, don't you?' 'Upon my word it is the best remedy I know of.' 'What springs would you recommend, Doctor?' 'Any will do, Madam, where you can get plenty of soap and water.'" 97.

We dare say that many of our readers are familiar with the well-known squib on Lettsom.

" When any sick to me apply,
I physicks, bleeds and sweats' em;
If after that they choose to die,
What's that to me,

I. LETTSOM.

The following answer to this *jeu d'esprit*, was written by his friend Sir M. Martin:—

" Such swarms of patients do to me apply,
Did I not practice, some would surely die;
'Tis true, I purge some, bleed some, sweat some,
Admit I expedite a few, still many call

I. LETTSOM.

*Sir Richard Croft* married a daughter of Dr. Denman, gained, or materially added to, his reputation by attending the Duchess of Devonshire in her confinement, and lost his reason as well as his life by attending the Princess Charlotte in her's.

It was proved, says the Editor of these Sketches, at the inquest held on his body, that since the death of the Princess Charlotte, he had never been free from melancholy and abstraction; and on Dr. Thackeray's asking after one of his patients, he replied, 'That he would give five hundred guineas if it were over, rather than have to attend her." On the night of his suicide, he exclaimed to his servant, who complained of being unwell, " What is your agitation compared to mine?" And a few days before, he struck his forehead with much violence, in the presence of Mr. Hollings, and abruptly said to him, " Good God! what will become of me?" His conduct, with reference to the Princess, was much censured at the time; but Dr. Baillie stated that every thing was done by Sir Richard Croft that skill and humanity could suggest. The fatal issue of the case filled his mind with despair, and alarmed his friends for the safety of his intellect. He was heard frequently to ejaculate to himself, " I shall go mad—I shall end my life in a mad-house—I am ruined—Who will place any confidence in me?" As he was driving in his carriage to the Princess when she was taken ill, he observed to a member of his family, " This case will either make me, or ruin me." Poor Sir Richard! he appears to have had a presentiment that *something* was about to happen which would seriously affect his prospects in life. It is said that a few days before he attended his illustrious patient, he fancied he saw the Princess clad in white, glide through his bed-room. Sir Richard was a man of fine and honourable feelings, and was beloved by all who knew him.

*Cheselden* came before the public at an earlier period of life than almost any other in the long list of professional excellence. He was a fellow of the Royal Society at twenty-one years of age, and at twenty-two gave lectures on surgery! Living in an intellectual era, he was the friend and companion of it's master spirits. Pope often dined with him and thus writes of him. "As soon as I had sent my last letter, I received a most kind one from you, expressing great pain for my late illness at Mr. Cheselden's. I conclude you were eased of that friendly apprehension in a few days after you had despatched yours, for mine must have reached you then. I wondered a little at your question, who Cheselden was. It shows that the truest merit does not travel so far any way as on the wings of poetry: he is the most noted, and most deserving man in the whole profession of chirurgery; and has saved the lives of thousands by his manner of cutting for the stone."

Many of our readers have heard one part, at least of the following anecdote:—

"Notwithstanding the extensive practice he had been engaged in, he always, before an operation, felt sick at the thoughts of the pain he was about to inflict; though during its performance, his coolness and presence of mind never forsook him. In alluding to this feeling, Moraud relates an anecdote of a French surgeon, who, on visiting the hospital, expressed great surprise at witnessing such an evidence of weakness, as he considered it, on the part of so famous a surgeon. After the operation was over, the visiter was invited by Cheselden to accompany him to the fencing school, whither he was going to see a sparring match; but here the tables were completely turned, for no sooner did the contest begin, than the stranger turned pale at the sight, and was obliged speedily to betake himself to the open air." 114.

A *relation* of his was condemned to be hanged. Cheselden proposed, if the King would pardon him, to take out the drum of the prisoner's ear, in order to try what effect it would have; and if it succeeded, the experiment was to be repeated on Lady Suffolk. The man was pardoned, but the operation was never performed! It is a good thing for a felon to be related to a great Doctor.

Cheselden lost £1700. by his work upon the bones. Douglas, who wrote a peevish critique upon it, called Belchier up one morning at six o'clock, when the work was published, and asked him if Cheselden was mad, by saying that he had not room enough to write more on the bones. "What!" said he, "could he not get more paper."

*Percival Pott* "was originally intended for the church, in which he had considerable prospect of preferment; but he resisted the importunities of his friends, having manifested, in early life, a strong propensity for the profession to which he was destined in after life to become so bright an ornament. In 1736, Pott commenced practice in Fenchurch-street. In 1749, he was appointed one of the principal surgeons of St. Bartholomew's Hospital. At this period the state of the science of surgery was still very imperfect, notwithstanding some sensible and ingenious men had published observations which had enlightened and improved it. Still the maxim, '*Dolor medicini doloris,*' remained unrefuted; the severe treatment of the old school, in the operative part of surgery continued in force; the first principles of the science, the natural process and powers of healing, were either not understood, or not attended to; painful escharotic dressings were continually employed; and the actual cautery was in such frequent use, that, at the times when surgeons visited the hospital, it was regularly heated and prepared in the wards of the hospital and in the presence of the patients, as a part of the necessary apparatus. Mr. Pott lived to see these remains of barbarism set aside, and a more humane and rational plan, of which he was the originator, universally adopted.

An accident which Pott met with made him an author. As he was riding he was thrown from his horse, and suffered a compound fracture of his leg, the bone being forced through the integuments. Conscious of the danger attendant on fractures of this nature, and thoroughly aware how much they may be encreased by rough treatment or improper position, he would not suffer himself to be moved until he had made the necessary depositions. He sent to Westminster for two chairmen to bring their poles; and patiently lay on the cold pavement, it being the middle of January, until they arrived. In this situation he purchased a door, to which he made them nail their poles. When all was ready, he caused himself to be laid on it, and was carried through Southwark, over London-bridge, to Watling-street, where he was then living—a tremendous distance in such a state! It was during his confinement in consequence of this accident, that he planned and partly executed his celebrated treatise on Ruptures." 117.

Pott went a philosophical way to work in collecting the materials for his treatise upon Injuries of the Head. He was much engaged in practice at this time; and in order to amass many facts bearing upon the subject of his treatise, he was in the practice of visiting, almost daily, every hospital in London; and entered into a correspondence with the most eminent continental surgeons. "I must have facts," he would say: "what are mere opinions worth; any man may give an opinion, but it is not every mind that is qualified to collect and arrange important facts?"

Pott was very kind to medical men in distress. At one period he had three surgeons who had been reduced in circumstances, living in his own house, until he could find means by which they could earn an independent livelihood. He was most kind and attentive to the patients in the hospital: in particular cases he was not satisfied with seeing them at the ordinary periods, but he visited the hospital several times during the day. On one occasion he had performed a complicated and dangerous operation. He had seen the patient three times during the day; and during the following night he dreamed that the assistant at the hospital had forgotten to administer a particular medicine that he had ordered, and from which he had anticipated much good effect. He could not remain easy whilst this impression was on his mind. He rose from his bed, dressed himself, and in the middle of the night, without disturbing any of the family, went to the hospital, gained admission, had the house-surgeon summoned to his side, in order to satisfy himself that his instructions had been punctually obeyed. To his great astonishment he found that the medicine had not been exhibited! Pott always declared that his dream had saved his patient's life.—Although a great surgeon and skilful operator, Pott was nervous prior to performing any difficult operation. He never allowed a day to pass without dissecting some portion of the human body. He considered it the sacred duty of every surgeon who might be called upon to operate upon the living, to be always operating upon the dead.

The person of Mr. Pott was elegant, though less than the middle size; his countenance animated and expressive; his manners and deportment were graceful; and his remarkable vigour and activity seemed unabated by age. On the last day of his illness he observed, "My lamp is almost extinguished; I hope it has burned for the benefit of others."

*James Heaviside* is generally known for his museum. He received an easy fortune on his father's death. Yet he practised his profession with assiduity and success. He never left London for recreation; and was always forthcoming, whenever an emergency required his presence. His domestic staff was well appointed, and his zealous aid-de-camp, who was his valet for thirty years, never failed to produce his master at any hour of the day and night; which, perhaps, may furnish us with a reason why he was so frequently called in to cases of accidents, and consequently so often figured as a witness in the courts of justice. "Egad," said Jekyl one day, "we never have a *homicide*, or a *suicide*, or any other *cide*, without a *Heaviside!*"

The principal event in his life arose out of his professional attendance at the fatal duel between Colonel Montgomery and Captain Macnamara, in 1802. For this he was committed to Newgate, and during a whole fortnight subjected to much pain and anxiety. His friends, Messrs. Erskine and Garrow, Mr. afterwards Baron Wood, and others, volunteered their aid; but still, the dread of the capital punishment, and with it the forfeiture of his property, made his situation seriously uncomfortable. In this dilemma, his brokers, Messrs. Johnson and Langden, sold out his stock, which, together with the rest of his possessions, was conveyed to a third party in safety; and to the credit of these gentlemen it should be told, that, though the amount must have been an object to be desired, yet, under the circumstances of the case, they transacted the business without the charge of a single farthing. In the end, the grand jury ignored the bill against Mr. Heaviside.

### Literary and Scientific Medical Men.

The author of the sketches remarks that:—"it is a singular anomaly in the constitution of medical affairs, that a man's success as a practitioner is often in an inverse ratio to his scientific attainments! This is in a great measure to be attributed to the circumstance of the public believing that scientific knowledge is incompatible with practical skill or tact, as well as to the encouragement which the great give to ignorant and shallow pretenders to a knowledge of medicine.

It may be true, to a certain extent, that he who devotes his whole time to the study of any one of the sciences which are embraced in the study of medicine, to the exclusion of all practical investigations, is not pursuing a course best adapted to make him an acute practitioner; yet it must not be forgotten that even in the minor details and minutiæ of practice, a man of science may exhibit his superiority over the merely matter-of-fact and experienced physician or surgeon."

And, after some other observations, he adds:—"In no science so much as in medicine is a reference to principles of so much consequence, for no where does the mere sequence of events, the *post hoc propter hoc* mode of argument, so often lead into error. 'Without principles,' says Dr. Cullen, 'deduced from analytical reasoning, experience is a useless and a blind guide.'

From the preceding observations the reader will perceive the immense superiority which the man of science has over the practitioner who has nothing to guide him, but undigested and unarranged facts. The former is seldom or never at a loss, the latter is always floundering and uncertain in his practice."*

* "He who follows certain arts or practical rules, without a knowledge of

We are not maintaining that the man of science, without experience is sufficient to constitute a good practitioner; but we do avow that in proportion as a medical man has a knowledge of the principles of medicine, so does he become qualified to grapple successfully with the various alarming diseases which will demand attention in the course of his practice."

We cannot be accused in fairness of undervaluing literary or scientific attainments; nor have we ever been advocates for empirical and routine practice. But it would be idle to maintain that practical attainments are consistent with any amount, how great soever, of devotion to literature or collateral science. There certainly is a point beyond which such devotion cannot be pushed without distracting the attention from practical pursuits, and interfering with the acquisition of practical knowledge. What that amount is it would be hard to say. We doubt if it is an invariable quantity. It depends very much on the temper of mind of the physician—if that is exact, if it has the qualities which constitute it "practical," then literature or science may be dived into pretty deeply, without much mischief resulting.

To make a good physician or surgeon, it is necessary that a man should understand thoroughly the principles of medicine, and should possess the power of analyzing symptoms, as well as of quickly seizing the important ones. Acquaintance with the science may be got by study—the faculty of deciphering the language of disease can only be obtained at the bed-side. If men will spend their time in their libraries or their laboratories, we do not see how they can acquire a knowledge of disease, or how they can hope to be expert at curing it. If such men complain of the blunders of the public, they are certainly unreasonable. The public care little (why should they?) for the *science* of medicine. What they want is to be relieved or cured. A man with pneumonia does not ask for a poem, or a quotation from Hippocrates, or a lecture on electro-magnetism; he is suffering, and claims relief, in danger, and implores for succour. Whoever does this best, is, to all intents and purposes, the best physician; and to do this well implies as much devotion to a special train of observation, as to excel in chemistry requires *it's* studies, or in natural philosophy *it's*. Practical men in our profession are such as, for the most part, avail themselves of the collateral science of others. In medicine, as in other things, the inventors are not always the best to apply inventions.

To return to our author:

"It is lamentable," says he, "to think how little encouragement is offered to medical men, in this country, to pursue with ardour their researches into the dominions of science.

In England the members of the medical profession are compelled to devote nearly the whole of their attention to the practical part of medicine. Very few men commence the study of physic with a view of attempting to enlarge its scientific boundaries. Lucrative practice is the natural and great aim of most who enter the profession." 183.

---

the science on which they are founded, is the mere artizan or the empyric; he cannot advance beyond the practice-rules which are given him, or provide for new occurrences and unforeseen difficulties."—*Abercrombie* "*On the Intellectual Powers,*" *p.* 18.

"In England, if a man obtains eminence on account of a scientific discovery, or in consequence of his great practical skill, he may be honoured with knighthood; and if he has great interest at court, he may succeed in being made a baronet; but these honours (?) come when he is least in want of them. A man who has already obtained eminence in his profession stands in but little need of an empty-sounding title, which at the most can only please his vanity, without adding one iota to his fame and reputation.

They manage things better in France. In that country the sciences and scientific men are encouraged and made comfortable: liberal allowance is provided for every member of the academies; and it is calculated that not less than one hundred thousand pounds is annually expended in pensions to men of science, of whose services, in various ways, ministers avail themselves. In France, titles of nobility and crowns of honour and merit are abundantly bestowed, and with the happiest effect. The late Dupuytren had the dignity of baron conferred upon him; and the same honour was paid to Larrey. The present king conferred the legion of honour upon Biett, Lallemand, Andral, and Chomel; thus demonstrating at once his respect for the science, and the professors of medicine." 186.

The fact is, as we have observed, that the public will always patronise the art—some other patron must be found for science. It seems strange that this has escaped our legislature and executive. Of all governments in Europe ours does least for science. Lord Melbourne, in his famous display with Faraday, said that pensions to scientific men were humbug! In this, we see the vulgarity of the tradesman, and the contemptible narrow-mindedness of the aristocratic leader of a faction. This same lord would cram the public offices with his relations or his parasites, and directly or indirectly pension every pimp to his party.

Unless something is done to encourage the *haute science* in this country, we shall soon be in the rear of civilized nations. The public is made up of individuals, and individuals will only purchase what is directly useful to them. Science is not of immediate utility, although it is to science that the usefulness of art is due. The public pay the practical man—the practical man owes much of his power to the scientific man—the scientific man is not encouraged, because society contains no class that immediately requires his article—and the consequence will be that science will first flag, and art afterwards. The government must step forward, and here, as abroad, erect professorships, by which science may get its bread. It cannot be tolerated that, in this wealthy country, government assistance, in other words the money of the nation, should be squandered on those only who assist the purposes of faction; and superciliously refused, or dealt with a niggard hand, to those who in the scale of human intellect are far superior to the political centurions who lord it over them.

"*John Locke* was originally intended for the profession of physic. He took a Bachelor's degree in medicine, in February 6, 1674, and was actually practising at Oxford, when accident brought him acquainted with Lord Ashley, afterwards Earl of Shaftesbury and Lord Chancellor. His lordship being advised to drink the mineral waters at Acton, for an abscess in his breast, wrote to Dr. Thomas, a physician at Oxford, to procure a quantity of those waters to be ready at his coming there. Dr. Thomas being called away by other business, easily prevailed with his friend Locke to undertake the affair, who happening to employ a person who disappointed him, he was obliged to wait on his lordship to apologize. Lord Ashley, with his usual politeness, received him with great civility, and was satisfied with his apology; and being much pleased with his conversation, detained him to supper, and engaged him to dinner the next day, and even to drink the waters, as he was desirous of enjoying his company. Lord Ashley not finding much benefit from the waters, Locke opened the abscess in his breast, and, it is said, saved his lordship's life." 186.

There is rather a curious letter from Locke to Sir Hans Sloane.

"Dear Sir,—I have a patient here sick of the fever at this season; it seems not violent; but I am told 'tis a sort that is not easily got off; I desire to know of you what your fevers in town are, and what methods you find most successful in them. I shall be obliged by your favour, if you will give me a word or two by to-morrow's post, and direct it to me, to be left at Mr. Harrison's, in the Crown, at Harlow.

I am, Sir,

Your most humble servant,

J. LOCKE."

Locke, wisely we think, turned to metaphysics and politics, and *cut* physic —*not* abscesses.

*Dr. William Hyde Wollaston* was educated at Caius College, Cambridge, and became a Doctor of Physic.

"Success, however, in the professors of medicine, is not at all times to be commanded, even by men of the highest attainment. Quackery and ignorance will thrive, while knowledge and honesty starve. And Dr. Wollaston, with all his cultivation of mind, and high professional advancement, failed to obtain that patronage from the public which he had a greater right to expect than many of his more successful contemporaries—being unable to stoop to those little arts of meanness and flattery, which are sometimes more successful in establishing a practice than learning and knowledge. Dr. Wollaston retired from the competition in disgust; and for the happiness of man, and interest of science, resolved never again to write a prescription, even for his father, but to devote the remainder of his life to the pursuit of chemical philosophy." 198.

We are by no means sure that Wollaston was qualified for practice. It seems dubious whether his repugnance to a little art was invincible. For instance:—

"Anecdotes are told respecting the resolute manner in which he uniformly resisted the intrusion of either friend or stranger into his workshop; among others, it is related, that a gentleman of his acquaintance having been left by his servant to ramble from one room to another, till he should be ready to see him, penetrated into the laboratory. The doctor, on coming in, discovered the intrusion; but not suffering himself to express all he felt on the occasion, took his friend by the arm, and, having led him to the most sacred spot in the room, said, 'Mr. P. do you see that furnace?' 'I do.' 'Then make a profound bow to it, for this is the first, and will be the last time of your seeing it.'" 199.

The fact is, that Wollaston had a keen eye for gold, and he was afraid of any man's taking a leaf out of his book. If this is not a very tradesman-like view of science, we should be glad to be informed what is. And a very good tradesman Wollaston was, for his fortune was considerable, amounting to £50,000 independently of an estate which he possessed in Sussex, and all of which was amassed by his own application and abilities. His most important discovery, the malleability of platinum, by a purifying instead of an alloying process, produced him, in the long run, about £30,000.

In a chapter on—

### Mad Doctors and Mad Houses, we find some particulars of the life of *Dr. Willis*, to one or two of which we may refer.

Willis, writes our author, was a student of Brazen-nose College, Oxford, where he graduated M.A. and took holy orders, and obtained some time afterwards the living of St. John's, Wapping. Having, however, previously to his ordination, paid some attention to physic, he made his knowledge of that art so useful to his neighbours, that the doctors of Thetford, in Lincolnshire, where he resided, were greatly incensed at his success, and threatened him with a prosecution. This induced him to procure a medical diploma, which was granted him by the University of Oxford, in 1759, and shortly afterwards he became celebrated for his treatment of Insanity. He was called in to George the III. at the suggestion of Dr. Addington. He assured the ministers and the queen that the king would speedily recover. Unpleasant altercations took place among the physicians. Willis was strongly suspected of circulating reports rather gratifying to the minister than consonant to truth. He regularly sent to him every night a particular message, and generally by his son. On the 16th, whilst the subject of the king's illness was being debated in the House of Commons, Mr. Pitt and his friends declared that that evening, at eleven o'clock, the son of Dr. Willis arrived at the Treasury, with the satisfactory account that a happy change had taken place; and that Dr. Willis considered it as a certain indication of the king's speedy recovery.

When Sir N. Wraxall saw Willis not long after the king's restoration to health, he says that he (Willis) seemed to be exempt from all the infirmities of old age; and his countenance, which was very interesting, blended intelligence with an expression of placid self-possession. When summoned to attend the king, he readily obeyed, but, at the same time, he frankly informed her majesty, that if she expected any benefit to accrue from his attendance, he must be allowed to exercise the same authority which he should do over the meanest individual submitted to his control. He brought his own keepers with him, and dismissed every body who was in attendance upon his Majesty. The furniture was changed, and every thing was done to deprive his patient of old associations.

A proof which he displayed of his empire over his patient excited great amazement, not unmixed with alarm, as well as admiration. The king had not undergone the operation of shaving during more than five weeks, nor would submit to have it performed, yet expressed a strong desire to shave himself. Willis gratified him in this wish. "Your Majesty," said he, "is desirous to get rid of your beard. You shall have a razor given to you for the purpose." He instantly put the instrument into the king's hand, who went through the process with perfect success, Willis governing him by the eye throughout the whole performance.

Dr. Warren, in his examination before the Parliamentary committee, stated that when he asked Dr. Willis how he could trust the king with a razor, he said, "I shuddered at what I had done."

The celebrated Edmund Burke asked Willis how he should have acted if his Majesty had been seized suddenly with frenzy, while the razor was in his hand. Upon this Dr. Willis desired two vivid lights to be placed between the great orator and himself, and exclaimed, "There now, I should look at him thus," darting, at the same time, such a look at Burke from his appalling eye, as made him recoil with affright. This mode of looking at a maniac, he observed, would cause him to quail more effectually than chains or manacles.

We believe that we must now shut this gossiping volume. We have already remarked that we would not vouch for the correctness of many of the anecdotes: we *would* vouch for the *incorrectness* of some of them. They seem to have been got together, perhaps in a hurry, certainly without much care or discrimination. The accounts of living medical men are, for the most part, ridiculously inaccurate. The work carries with it some marks of an extra-professional hand, and report has assigned it to the clever author of "the Diary of a Physician." If that be so, we look in vain for the powerful writing which that Diary contained, though the same vein of extravagance runs through both. With all the defects which criticism can point out, the blunders in matter of fact, the distorted view of professional life and professional success, there is still something to instruct and a good deal to amuse in these volumes.

We cannot conclude without again recommending our readers not entirely to neglect the biography and history of their profession. It is astonishing how ignorant the majority of medical men are of both. Yet in each there is room for useful study, and each furnishes important lessons to whoever chooses to learn them.

We have not on this occasion adverted particularly to the work of Mr. Pettigrew. We have already recommended it; and on a future occasion we shall bring up our arrears of notice of it. Mr. Pettigrew deserves the support of the profession, and no medical library, private or public, should be without his volume.

